# Templated α-Synuclein Inclusion Formation Is Independent of Endogenous Tau

**DOI:** 10.1523/ENEURO.0458-20.2021

**Published:** 2021-06-16

**Authors:** Lindsay E. Stoyka, Casey L. Mahoney, Drake R. Thrasher, Drèson L. Russell, Anna K. Cook, Anner T. Harris, Ashwin Narayanan, Tiara P. Janado, David G. Standaert, Erik D. Roberson, Laura A. Volpicelli-Daley

**Affiliations:** Center for Neurodegeneration and Experimental Therapeutics, University of Alabama at Birmingham, Birmingham, AL 35294

**Keywords:** dementia, Lewy, neurodegenerative, Parkinson’s disease, synuclein, tau

## Abstract

Synucleinopathies including Parkinson’s disease (PD) and dementia with Lewy bodies (DLB) are characterized by neuronal intracellular inclusions of α-synuclein. PD dementia (PDD) and DLB are collectively the second most common cause of neurodegenerative dementia. In addition to associated inclusions, Lewy body diseases (LBDs) have dopaminergic neurodegeneration, motor defects and cognitive changes. The microtubule-associated protein tau has been implicated in LBDs, but the exact role of the protein and how it influences formation of α-synuclein inclusions is unknown. Reducing endogenous tau levels is protective in multiple models of Alzheimer’s disease (AD), tauopathies, and in some transgenic synucleinopathy mouse models. Recombinant α-synuclein and tau proteins interact *in vitro*. Here, we show tau and α-synuclein colocalize at excitatory presynaptic terminals. However, tau heterozygous and tau knock-out mice do not show a reduction in fibril-induced α-synuclein inclusions formation in primary cortical neurons, or after intrastriatal injections of fibrils at 1.5 month or six months later. At six months following intrastriatal injections, wild-type, tau heterozygous and tau knock-out mice showed a 50% reduction in dopamine neurons in the substantia nigra pars compacta (SNc) compared with mice injected with α-synuclein monomer, but there were no statistically significant differences across genotypes. These data suggest the role of tau in the pathogenesis of LBDs is distinct from AD, and Lewy pathology formation may be independent of endogenous tau.

## Significance Statement

Variations in the *MAPT* H1 haplotype are associated with Parkinson’s disease (PD), but it is possible that other genes within the H1 haplotype play a role in PD etiology. *In vitro* studies show α-synuclein and tau interact, leading to synergistic fibrillization. α-Synuclein and tau can co-exist in Lewy bodies. Tau reduction is protective in models of Alzheimer’s disease (AD) and tauopathies and has been suggested as a therapeutic strategy for PD. Here, we show reduction of endogenous tau does not influence formation of templated α-synuclein inclusion formation or loss of dopamine neurons, suggesting that therapeutics directed to tau for PD may be more complicated than tau reduction.

## Introduction

Neuronal synucleinopathies include Parkinson’s disease (PD), PD dementia (PDD), and dementia with Lewy bodies (DLB), referred to as Lewy body diseases (LBDs). LBDs are characterized by intracellular inclusions composed mostly of α-synuclein, found in the soma, termed Lewy bodies, and axons, called Lewy neurites (Spillantini et al., 1997, 1998). In addition to localizing to brain areas important for movement such as the substantia nigra pars compacta (SNc), Lewy pathology is found in the cortex and limbic brain regions where it may contribute to cognitive and psychiatric symptoms (Mattila et al., 1998; Braak et al., 2003, 2005; Jellinger, 2009; Del Tredici and Braak, 2013; Hall et al., 2014; Irwin et al., 2017; Jellinger and Korczyn, 2018).

Although the majority of LBDs are idiopathic disorders, genetic factors are implicated in the pathogenesis and risk factors of these diseases. The gene for α-synuclein, *SNCA*, has the highest population attributable risk (PAR) in genetic studies of PD risk, and mutations or multiplications in this gene cause early-onset familial PD (Blauwendraat et al., 2020). A strong association of variations in *MAPT* 17q.21.31 locus H1 haplotype also occurs with PD (Blauwendraat et al., 2020). However, many genes are located within this locus, and it remains unclear whether *MAPT* alone is the primary culprit.

The protein product of *MAPT*, microtubule-associated protein tau, forms hyperphosphorylated neurofibrillary tangles (NFTs), one of the main characteristics of Alzheimer’s disease (AD). The role of tau in synucleinopathies is poorly understood. The presence of both Lewy pathology and NFTs composed of tau in the cortex are the strongest correlate of cognitive decline in PDD (Irwin et al., 2017; Coughlin et al., 2019). *In vitro* experiments demonstrate the microtubule binding domain of tau directly interacts with the C terminus of α-synuclein, promoting synergistic fibrillization of both proteins (Giasson et al., 2003; Dasari et al., 2019). Electron microscopy shows that tau aggregates are found in the same neuron as those harboring Lewy pathology (Duda et al., 2002; Ishizawa et al., 2003). These data suggest that an interaction of α-synuclein and tau may contribute to the etiology of PD.

Reduction of tau is protective in models of AD and other tauopathies reducing cognitive deficits, seizure susceptibility, and premature mortality (Roberson et al., 2007; DeVos et al., 2017, 2018). The role of tau reduction in PD and LBDs has been more challenging to elucidate. In mice overexpressing human α-synuclein, absence of tau rescues memory deficits as tested by Barnes maze, contextual fear conditioning, and novel object recognition (Singh et al., 2019). Antibodies to oligomeric tau reduce memory defects and Lewy-body like pathology in mice overexpressing the A53T mutant form of α-synuclein (Gerson et al., 2018). In contrast, absence of tau did not prevent templated spreading of misfolded α-synuclein, or dopamine neuron loss and associated motor deficits in toxin-induced and transgenic models of human-α-synuclein overexpression (Morris et al., 2011; Gratuze et al., 2019; Jiao et al., 2020; Bassil et al., 2021). The effect of tau reduction in PD models, therefore, differs from AD models, and further characterization is necessary.

Small seeds of fibrillar α-synuclein corrupt endogenous α-synuclein to form inclusions biochemically and morphologic resembling those found in PD and DLB (Volpicelli-Daley et al., 2011; Luk et al., 2012; Paumier et al., 2015; Froula et al., 2019; Sorrentino et al., 2019). Use of intrastriatal injections of recombinant α-synuclein fibrils recapitulates Lewy-like pathology in brain regions critical to LBDs such as the SNc, cortex and amygdala. Because fibrils induce the formation of inclusions from endogenously expressed α-synuclein, this model allows us to use tau heterozygous and tau knock-out mice to determine the role of tau reduction in template α-synuclein inclusion formation.

Here, our goal was to investigate whether tau reduction influences templated α-synuclein inclusion formation. First, we show that α-synuclein and tau colocalize at the presynaptic terminal of excitatory neurons. However, neither tau heterozygous nor tau knock-out mice show reductions in seeded α-synuclein inclusions or loss of dopamine neurons in the SNc. Thus, reduction of endogenous tau does not appear to prevent the templated formation of α-synuclein inclusions.

## Materials and Methods

Unless otherwise noted, all materials were purchased from Fisher Scientific.

### Animals

All animal protocols were approved by our University’s Institutional Animal Care and Use Committee. Mice were on a 12/12 h light/dark cycle and had *ad libitum* access to food and water. Both male and female mice were used in this study. C57BL6/J mice were obtained from Jackson Labs. Tau wild-type, tau heterozygous, and tau knock-out mice were a generous gift from Erik Roberson, University of Alabama at Birmingham (full description in preparation). Tau*^flox/flox^* mice contained loxP sites flanking exon 1, similar to the constitutive tau knock-out mice generated by deleting exon 1 (Dawson et al., 2001). The mice were crossed with flipase transgenic mice, which removed the neomycin cassette, then crossed to β-actin-Cre mice to generate the tau knock-out line. The mice were created in C57BL/6 ES cells and maintained on a congenic C57BL/6J background.

### Preparation of fibrils

Fibrils were generated as described previously (Stoyka et al., 2020). Mouse α-synuclein was purified in *Escherichia coli* using a Pierce LAL high-capacity endotoxin removal resin to minimize endotoxin. The concentration of monomeric α-synuclein was measured by absorbance at 280 nm with an extinction coefficient of 7450 M^−1^ cm^−1^. Fibrils were generated by incubating monomeric α-synuclein (300 μm) in 150 mm KCl, 50 mm Tris-HCl at 37°C with constant agitation for 7 d (Bousset et al., 2013). After the seventh day, fibrils were isolated from remaining monomer by centrifugation for 10 min at 13,200 rpm and resuspended in half the initial volume of buffer. Five μl of fibrils were incubated for one hour with 8 m guanidinium chloride to dissociate the fibrils into monomer, the concentration of α-synuclein was measured, and remaining fibrils were diluted to a final concentration of 300 μm; 22 μl aliquots were stored at −80°C. On the day of injection, the fibril aliquots were thawed and sonicated using a Qsonica 700W cup horn sonicator system at 80% amplitude for two rounds of 15 min sonication at 3 s on/2 s off, held at 15°C for a total sonication time of 30 min. After 15 min of sonication, droplets on tube walls were gently scraped down into solution using a pipette tip. Size of sonicated fibrils was measured to ensure proper sonication using dynamic light scattering on a DynaPro NanoStar Dynamic Light Scattering Detector (Wyatt Technology). Although this method is optimized for round particles and only approximates the sonicated fibrils, this method ensures sufficient fragmentation of the fibrils (Froula et al., 2019), which is essential for the fibril model to induce sufficient formation of α-synuclein inclusions. The average size of sonicated fibrils was under 60 nm. Sonicated fibrils were kept at room temperature during injections. We have found the fibril fragments generated using this procedure remain stable for at least 3 d at room temperature. Before injection of monomeric α-synuclein, protein was thawed on ice, spun at 20,000 × *g* at 4°C, and kept on ice until injected.

### Primary neuron cultures

Neurons were prepared as described previously (Volpicelli-Daley et al., 2014a,b). For all experiments, heterozygous mice for the tau knock-out gene were bred to give littermate pups of all three genotypes. Upon dissection, tail clippings were taken to confirm genotype of pups. Briefly, cortices were dissected from embryonic [embryonic day (E)16–E18] mice in Hibernate E, digested with papain in HBSS with 10 mm HEPES, 1 mm sodium pyruvate, and 1% penicillin/streptomycin, then titrated and plated in Neurobasal media (Invitrogen) with B27 (Invitrogen), GlutaMAX (Invitrogen), penicillin/streptomycin, and 10% fetal bovine serum. Twelve hours later, the media was changed to Neurobasal/B27/GlutaMAX without fetal bovine serum or penicillin/streptomycin. Neurons were plated at a density of 5 × 10^5^ cells per plate for 35-mm dishes or 1 × 10^5^ cells per coverslip.

### Proximity ligation assay (PLA)

Association between tau and α-synuclein was examined using PLA (DuoLink PLA kit, Sigma) with an anti-mouse PLA probe plus and anti-rabbit PLA probe minus. Primary antibodies were monoclonal mouse antibody to the C-terminal of α-synuclein (SYN202, BioLegend, RRID: AB_2734606) and rabbit polyclonal antibody to the microtubule binding domain of tau (A0024, Dako, AB_10013724). DuoLink kit was used according to the manufacturer’s instructions. Briefly, cortical cultures at day *in vitro* (DIV)7–DIV10 were fixed for 30 min in 4% paraformaldehyde (PFA)/4% sucrose in PBS and permeabilized, then blocked in 5% normal goat serum in PBS and incubated with primary antibodies overnight at 4°C. After three rinses, coverslips were incubated with PLA probes in a humidified chamber for 1 h at 37°C. Probes were then ligated and amplified using included polymerase, ligase, and diluents. Supplied wash buffers were used according to manufacturer’s recommendation, and coverslips were mounted with supplied Duolink mounting medium. Provided mounting medium contained DAPI and coverslips were sealed using clear nail polish.

### Expansion microscopy

Expansion microscopy was performed as described (Chen et al., 2015; Asano et al., 2018; Wassie et al., 2019). Three-month-old C57BL/6 mice were transcardially perfused with 2% acrylamide in PBS followed by 4% PFA/30% acrylamide in PBS. Brains were then postfixed in the 4%PFA/30% acrylamide solution for 12 h and then stored in PBS at 4°C. Brains were sectioned coronally at 50-μm thickness on a Leica VT1000S vibratome. Sections were incubated for 30 min at 4°C in gelling solution of 86.6% monomer solution (prepared on ice, 0.38 g/ml sodium acrylate, Sigma; 0.5 g/ml acrylamide, Sigma; 0.29 g/ml NaCl; 1× PBS), 7.4% crosslinker solution (0.2 g/ml *N*,*N*’-methylenebisacrylamide, Sigma), 2% initiator solution (10% ammonium persulfate), 2% accelerator solution (10% tetramethylethylenediamine), 2% inhibitor solution (0.5% 4-hydroxy-TEMPO). Sections were then incubated for a further 2 h at 37°C, which initiated polymerization of the gel. Sections were trimmed to regions of interest and denatured using a solution of 200 mm SDS, 200 mm NaCl, 40 mm Tris, pH 9 for 1 h at 37°C and 1 h at 95°C. Denaturation removed PFA-induced crosslinking and ensures equal expansion of biomolecules.

Following denaturation, samples were rinsed with 5× with PBS, 20 min each with gentle agitation at room temperature. Samples were blocked 2 h at room temperature with agitation, and incubated with primary antibodies at 0.001 mg/ml in blocking solution (5% normal goat serum/0.1% Triton X-100 in 1× PBS) for 48 h at 4°C. Antibodies included SYN202 (BioLegend, RRID: AB_2734606) and tau (A0024, Dako, AB_10013724), and VAMP2 (Synaptic Systems, RRID: AB_2619758). After primary antibody incubation, samples were rinsed 5×, 1 h, in 1× PBS at 4°C with constant agitation. This was followed by an overnight incubation at 4°C with secondary antibody at 0.004 mg/ml in blocking solution. Finally, samples were rinsed in PBS, 1 h each, followed by three 20-min washes in DI water to reach full expansion size (∼6× initial size). Imaging was performed immediately on completion of expansion; imaging was completed using a 40× water-immersion objective (Nikon CFI Apo λ S objective; MRD77410) on an inverted Nikon A1 SIM confocal microscope.

### Immunoblotting

At three months of age, tau mice were anesthetized using isoflurane and transcardially perfused using 0.9% saline, 10 U/ml heparin, and 0.5% w/v sodium nitroprusside. Brains were removed and the midbrain and forebrain were isolated. Using fine forceps, the midbrain and forebrain were dissected into distinct brain regions consisting of the cortex, striatum, hippocampus, and midbrain, which were flash frozen using liquid nitrogen and stored at −80°C. Samples were processed for Western blotting using 0.1% Triton X-100 in TBS and mechanical homogenization using a motorized tissue grinder. After lysing, samples were centrifuged for 10 min at 4°C 1000 × *g*. The supernatant was diluted into Laemmli buffer with 5% fresh dithiothreitol (DTT) added. Samples were boiled for 5 min and resolved on 4–20% gradient Mini-PROTEAN TGX Precast Protein Gels (Bio-Rad) and transferred to PVDF membrane (Millipore). The membrane was fixed using 0.4% PFA in PBS for 30 min and washed 3 times in TBS/Tween (TBST; 25 mm Tris-HCl, 137 mm NaCl, and 0.1% Tween 20). Blots were blocked using 5% non-fat dry milk in TBST for 1 h at room temperature and then incubated at 4°C overnight with primary antibodies to mouse monoclonal Tau46 (Cell Signaling, AB_10695394), mouse monoclonal vinculin (Bio-Rad, AB_2214389), or rabbit monoclonal α-synuclein (AbCam, AB_869971) in blocking solution. After incubation, blots were washed three times with TBST and further incubated for 2 h at room temperature in IgG H + L Cross-Absorbed Goat anti-mouse HRP (Invitrogen) or IgG H + L Cross-Absorbed donkey anti-rabbit HRP (Invitrogen) blocking solution. Following three rinses with TBST, blots were incubated in enhanced chemiluminescence Western Blotting Substrate (Thermofisher) for 1 min at room temperature and developed using ChemiDoc Touch Imaging System (Bio-Rad).

### Intrastriatal injection of recombinant α-synuclein fibrils

At three to four months of age, male and female tau wild-type, heterozygous, and knock-out mice were deeply anesthetized with vaporized isoflurane on a stereotactic frame. Mice were then injected with 2 μl (per side) of 300 μm sonicated fibrils or 300 μm monomeric α-synuclein (as control) bilaterally into the striatum. The coordinates for the striatum were +1.0 mm AP, ±2.0 mm ML, –26 mm DV. Solutions were injected at a constant rate of 0.5 μL/min; once injection was complete, the needle was left in place for 5 min and then slowly withdrawn. Between 9 and 10 mice per group were used, resulting in a total of 56 mice used for the study.

### Behavior testing

Behavior tests to assess motor and non-motor function were conducted either 1.5 or six months after injection as described previously (Stoyka et al., 2020). One week before testing began, mice were transported to the behavior core; mice were handled for 3 d before testing began. Additionally, mice were habituated to the testing room for one hour at the onset of each testing day. All mice underwent open field test, followed by fear conditioning. Mice had 1–3 d of rest between testing paradigms. The researcher conducting and analyzing the test was blinded to treatment and genotype. All apparatuses were cleaned with 70% ethanol or 2% chlorhexidine between trials.

#### Open field test

Mice were placed in an open field apparatus that was 43 × 43 cm with 30-cm-high walls. To prevent bias from external factors, the walls were opaque such that mice could not view the external environment and a white noise generator was turned on for the entirety of the testing day. The lighting in the room was the overhead fluorescent lights. Mice were allowed to explore for 10 min. Velocity, mobility, and percent time in center were recorded by Med Associates Activity Monitor.

#### Fear conditioning

Mice were placed in a novel environment and trained to associate a tone (conditioned stimulus; CS) with a mild foot shock (unconditioned stimulus, US). After 2 min in the novel environment, a continuous tone would play for 30 s, culminating in a 0.8 mA, 2-s foot shock. Thirty seconds later, the tone and shock would repeat; 1 min after the second shock, the training trial ended. Twenty-four hours later, mice were evaluated for contextual and cued fear conditioning. During contextual fear conditioning testing, mice were placed in the same environment as training, and freezing behavior was evaluated for 5 min. Three hours later, mice were exposed to a novel environment (using novel scents, visual cues, and enclosure) and the CS is re-presented, this time without the foot shock. FreezeFrame software was used to record sessions. Freezing behavior during training, contextual, and cued fear conditioning was hand scored by a researcher blinded to treatment and genotype. Freezing behavior was defined as previously described (Stoyka et al., 2020).

### Immunohistochemistry and immunofluorescence

At either 1.5 or six to seven months after injection of α-synuclein fibrils or monomer, mice were anesthetized with isoflurane and transcardially perfused using 0.9% saline, 10 U/ml heparin, and 0.5% w/v sodium nitroprusside followed by cold 4% PFA in PBS. Brains were postfixed in 4% PFA in PBS for 16 h at 4°C, then switched to 30% sucrose in PBS for 24–48 h for cryoprotection. Samples were stored at −80°C until serially sectioned at 40-μm thickness on a freezing microtome, then stored at −20°C in 50% glycerol, 0.01% sodium azide in tris-buffered saline (TBS). For immunohistochemistry, sections were rinsed four times in TBS and quenched in 3% H_2_O_2_ in TBS for 10 min. Following three rinses in TBS, sections were incubated in antigen retrieval solution (10 mm sodium citrate, 0.05% Tween 20, pH 6) at 37°C for 1 h, were rinsed three times in TBS, and blocked using 5% normal goat serum (Equitech-Bio Inc) with 0.1% Triton X-100 in TBS for 1 h at 4°C. After blocking, sections were incubated in primary antibody solution of rabbit polyclonal antibody to tyrosine hydroxylase (TH; Sigma) in 5% normal goat serum in TBS at 4°C overnight. After washes, sections were then incubated in Biotin-SP AffiniPure Donkey Anti-Rabbit IgG H&L (Jackson ImmunoResearch) in 5% normal goat serum in TBS for 2 h, followed by incubation with Avidin-Biotin Complex Peroxidase Standard Staining kit reagent for 1 h at room temperature. Sections were developed using ImmPACT-3 3’-diaminobenzidine (DAB; Vector Labs), sequentially dehydrated as clarified previously (Stoyka et al., 2020), and mounted on charged slides using Permount.

For immunofluorescence, sections underwent antigen retrieval, blocking, and primary antibody solutions as above. Primary antibodies were, SYN202 (BioLegend, RRID: AB_2734606) and tau (A0024, Dako, AB_10013724), and VAMP2 (Synaptic Systems, AB_2619758), or rabbit monoclonal to pSer129-α-synuclein (AbCam, AB_869973). pSer129-α-synuclein has been extensively characterized for staining mature Lewy-like aggregates (Delic et al., 2018). Following rinses, sections were incubated in appropriate Alexa Fluor-conjugated secondary antibodies (Invitrogen) diluted in blocking solution, rinsed and mounted on charged slides and coverslipped using Prolong Gold (Invitrogen).

### Fluorescent microscopy, aggregate analysis, and unbiased stereology

Sections were imaged using a Zeiss Axiovert.Z1 microscope for wide-field fluorescence, Olympus BX51 microscope for bright field, or a Nikon C2 confocal microscope. Expanded sections were imaged on a Nikon A1 SIM confocal microscope. The researcher capturing images was blinded to genotype and treatment group.

For Lewy-like aggregate analysis, a single image of each side of the region of interest in fibril-injected mice [dorsomedial prefrontal cortex (dmPFC), basolateral amygdala, hippocampus, and SNc] was captured at the approximate middle of the region rostral to caudal using the Nikon confocal microscope. For larger regions (PFC and hippocampus), a series of images were tiled together to create a “large field” image using Nikon Elements software. Care was taken to ensure representative images were matched across samples, with imaged sections at the following AP positions relative to bregma: +2.0 mm for PFC, −1.34 mm for basolateral amygdala, −1.82 mm for hippocampus, and −3.16 mm for SNc. As animals were injected bilaterally, right and left hemispheres were denoted, but both were quantified for all regions except the dmPFC. In the dmPFC, the hemispheres are not connected and therefore separated during coronal sectioning. As hemispheres could not be matched, only the left hemisphere was quantified. No differences between hemispheres were noted in any region. A researcher blinded to genotype outlined the region of interest and quantified Lewy-like aggregates using Fiji CellCounter. Both neuritic and somal aggregates were included during quantification. For analyses, hemispheres were evaluated separately, and then the average aggregate burden was binned for each sample.

Unbiased stereological analyses of TH neurons in the SNc were conducted using the Olympus BX51 bright field microscope, while using an optical fractionator probe (Stereo Investigator software, Stereology Resource Center) as previously described (Harms et al., 2013). Briefly, four to five sections covering the SNc from rostral to caudal were quantified, representing a total of 960 μm. The optical dissector height was 22 μm, and the distance between the counting frame was 50 μm × 50 μm, with a section thickness of 33 μm. The counting variability was measured with the Schmitz–Hof coefficient of error (CE; Schmitz, 1998) and was 0.06.

### Statistical analyses

Statistical analyses were performed using GraphPad Prism. Data are presented as mean ± SEM. Data were first tested for normality using the Shapiro-Wilk normality test. Normally distributed data were analyzed using an independent, unpaired *t* tests (for two groups), one-way or two-way ANOVA. Data that did not fit a normal distribution were analyzed using a Kruskal–Wallis test (for three groups; Table 1).

**Table 1:** Statistical table describing the figure, data, type of test and statistics results

Figure	Graph identification	Type of test	Statistics
Fig. 1*A*		Kruskal–Wallis test	*H*_(2)_ = 120.6, *p* < 0.0001
Fig. 1*B*		One-way ANOVA	*F*_(2,6)_ = 1.1, *p* = 0.39
Fig. 1*C*	DIV10	Two-way ANOVA	Interaction: *F*_(2,263)_ =1.360, *p* = 0.2584
	DIV18	Two-way ANOVA	Interaction: *F*_(2,237)_ = 6.956, *p* = 0.0012; treatment: *F*_(1,237)_ = 16.61, *p* < 0.0001; genotype: *F*_(2,237)_ = 44.00, *p* < 0.0001
Fig. 2*C*	Cortex, tau	One-way ANOVA	*F*_(2,6)_ = 151.0, *p* < 0.0001
	Cortex, synuclein	One-way ANOVA	*F*_(2,6)_ = 2.703, *p* = 0.1455
	Striatum, tau	One-way ANOVA	*F*_(2,6)_ = 110.5, *p* < 0.0001
	Striatum, synuclein	One-way ANOVA	*F*_(2,6)_ = 3.611, *p* = 0.0934
	Hippocampus, tau	One-way ANOVA	*F*_(2,6)_ = 73.06, *p* < 0.0001
	Hippocampus, synuclein	One-way ANOVA	*F*_(2,6)_ = 0.112, *p* = 0.8958
	Midbrain, tau	One-way ANOVA	*F*_(2,6)_ = 152.1, *p* < 0.0001
	Midbrain, synuclein	One-way ANOVA	*F*_(2,6)_ = 0.1251, *p* = 0.8846
Fig. 3*B*	PFC	One-way ANOVA	*F*_(2,24)_ = 0.2523, *p* = 0.7791
	Basolateral amygdala	One-way ANOVA	*F*_(2,25)_ = 1.368, *p* = 0.2731
	Dentate gyrus	Kruskal–Wallis test	*H* = 1.161, *p* = 0.3223
	CA1–CA3	Kruskal–Wallis test	*H* = 2.265, *p* = 0.5597
	SNc	One-way ANOVA	*F*_(2,24)_ = 2.119, *p* = 0.1421
Fig. 4*B*		Two-way ANOVA	Interaction: *F*_(2,38)_ = 0.5941, *p* = 0.5571; treatment: *F*_(1,38)_ = 61.12, *p* < 0.0001; genotype: *F*_(2,38)_ = 0.1630, *p* = 0.8502.
Fig. 5*A*	Velocity	Two-way ANOVA	One outlier removed interaction: *F*_(2,48)_ = 2.700, *p* = 0.0774; treatment: *F*_(1,48)_ = 3.248, *p* = 0.0778; genotype: *F*_(2,248)_ = 2.805, *p* = 0.0705.
	Distance	Two-way ANOVA	Interaction: *F*_(2,49)_ = 4.9, *p* = 0.01; treatment: *F*_(1,49)_ = 0.79, *p* = 0.38; genotype: *F*_(2,49)_ = 3.7, *p* = 0.03
	Percent time in center	Two-way ANOVA	Interaction: *F*_(2,49)_ = 0.7311, *p* = 0.4866; treatment: *F*_(1,49)_ = 0.1434, *p* = 0.7066; genotype: *F*_(2,49)_ = 0.7588, *p* = 0.4736
Fig. 5*B*	Training	Three-way ANOVA	Three-way interaction: *F*_(8,170)_ = 0.64, *p* = 0.7449; genotype × treatment: *F*_(2,170)_ = 0.9, *p* = 0.4075; genotype × time: *F*_(8,170)_ = 0.54, *p* = 0.8246; treatment × time: *F*_(4,170)_ = 0.16, *p* = 0.9583; treatment: *F*_(1,170)_ = 0.76, *p* = 0.385; genotype: *F*_(2,170)_ = 7.44, *p* = 0.0008; time: *F*_(4,170)_ = 21.57, *p* < 0.0001
	Contextual	Two-way ANOVA	Interaction: *F*_(2,46)_ = 0.7843, *p* = 0.4624; treatment: *F*_(1,46)_ = 0.003584, *p* = 0.9525; genotype: *F*_(2,46)_ = 1.826, *p* = 0.1725
	Cued	Two-way ANOVA	Interaction: *F*_(2,49)_ = 4.003, *p* = 0.0245; treatment: *F*_(1,49)_ = 1.171, *p* = 0.2844; genotype: *F*_(2,49)_ = 3.907, *p* = 0.0266
Fig. 6*B*	PFC	Independent *t* test	*t*_(14)_ = 1.8, *p* = 0.09
	Basolateral amygdala	Independent *t* test	*t*_(14)_ = 1.02, *p* = 0.32
	Hippocampus	Independent *t* test	*t*_(13)_ = 0.65, *p* = 0.52
	SNc	Independent *t* test	*t*_(14)_ = 0.53, *p* = 0.6
Fig. 6*C*	Velocity	Two-way ANOVA	Interaction: *F*_(1,27)_ = 3.1, *p* = 0.09; treatment: *F*_(1,27)_ = 0.5, *p* = 0.5; genotype: *F*_(1,27)_ = 1.4, *p* = 0.24
	Distance	Two-way ANOVA	Interaction: *F*_(1,27)_ = 0.13, *p* = 0.71; treatment: *F*_(1,27)_ = 0.47, *p* = 0.49; genotype: *F*_(1,27)_ = 0.2, *p* = 0.65
	Percent time in center	Two-way ANOVA	Interaction: *F*_(1,27)_ = 1.5, *p* = 0.23; treatment: *F*_(1,27)_ = 1.3, *p* = 0.26; genotype: *F*_(1,2)_ = 0.12, *p* = 0.72
Fig. 6*D*	Training	Three-way ANOVA	Interaction: *F*_(16,120)_ = 0.8, *p* = 0.67; genotype × treatment: *F*_(4,30)_ = 0.5, *p* = 0.71; time: *F*_(2,88)_ = 42.8, *p* < 0.0001
	Contextual	Two-way ANOVA	Interaction: *F*_(1,29)_ = 1.4, *p* = 0.23; treatment: *F*_(1,27)_ = 0.43, *p* = 0.51; genotype: *F*_(1,29)_ = 2.8, *p* = 0.11
	Cued	Two-way ANOVA	Interaction: *F*_(1,27)_ = 17.52, *p* = 0.0003; treatment: *F*_(1,27)_ = 4.5, *p* = 0.04; genotype: *F*_(1,27)_ = 3.9, *p* = 0.05
Extended Data Fig. 1-1		Kruskal–Wallis test	*H* = 0.6935, *p* = 0.7070
Extended Data Fig. 1-2*A*		Two-way ANOVA	Interaction: *F*_(2,268)_ = 7.2, *p* = 0.0009; treatment: *F*_(1,268)_ = 22.1, *p* < 0.0001; genotype: *F*_(2,268)_ = 1.678, *p* = 0.1886.
Extended Data Fig. 3-1		One-way ANOVA	*F*_(2,21)_ = 0.4911, *p* = 0.618

## Results

### Tau and α-synuclein interact in primary cortical neurons

**Figure 1.**
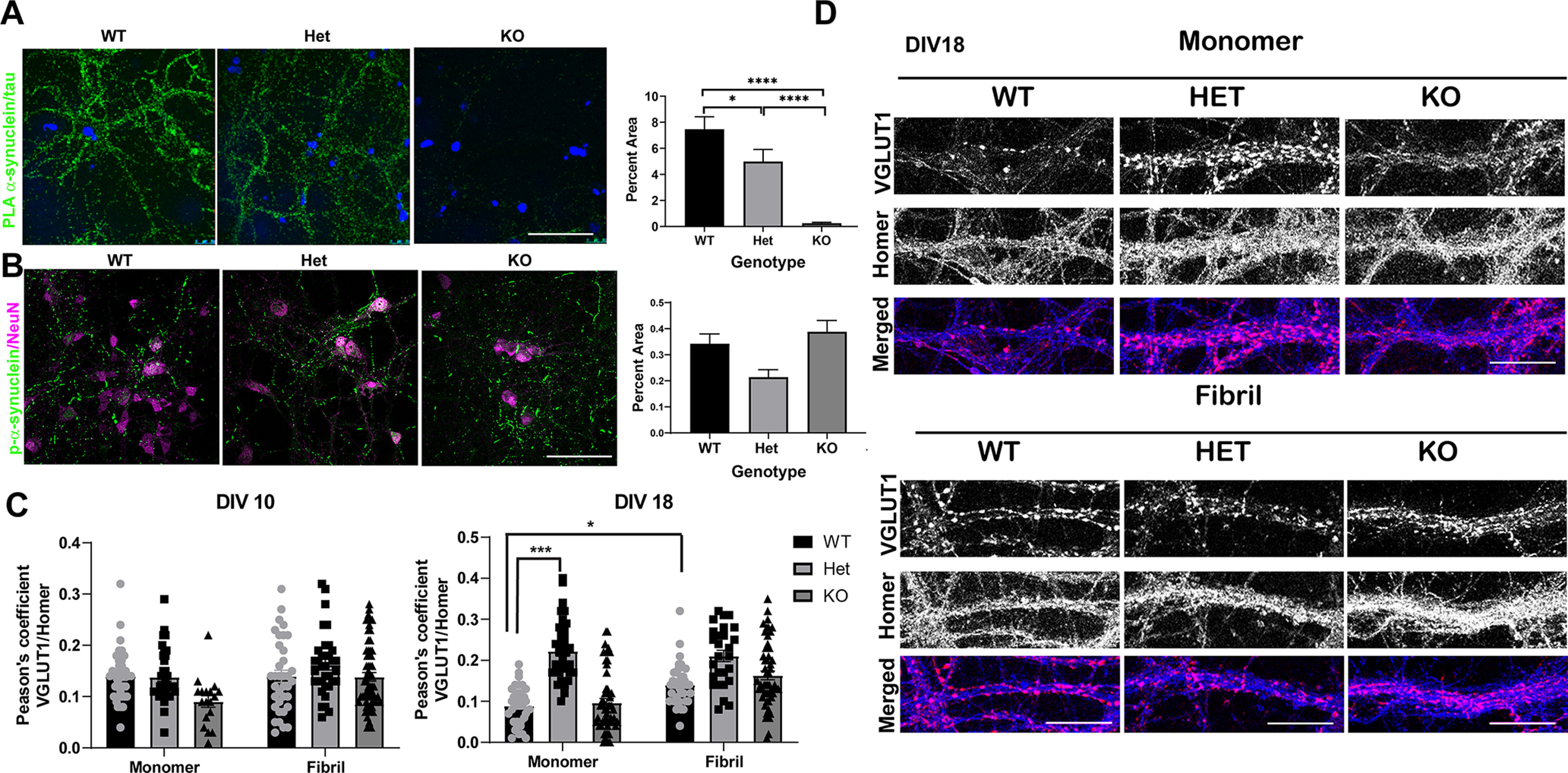
***A***, Primary cortical neurons at DIV14 from wild-type, tau heterozygous mice, or tau knock-out mice were fixed and labeled with antibodies to the C terminus of α-synuclein and the microtubule binding domain of tau. PLA was performed to detect an interaction between the two proteins. Images of 10 fields for each condition were captured using confocal microscopy. The percent area occupied by fluorescent signal was quantified using Fiji. The experiment was performed three times and data were analyzed with a Kruskal–Wallis test *H*_(2)_ = 120, *p* < 0.0001. Scale bar: 50 μm. ***B***, Primary cortical neurons were exposed to monomeric α-synuclein or α-synuclein fibrils 1 μg/mL at DIV7 from wild-type, tau heterozygous mice, or tau knock-out mice. Seven days later, neurons were fixed and labeled with antibodies to p-α-synuclein or NeuN to identify neuronal nuclei. The percent area occupied by fluorescent signal was quantified using Fiji. The experiment was performed three times. One-way ANOVA *F*_(2,6)_ = 1.1, *p* = 0.39, *p < 0.05, ***p < 0.001, ****p < 0.0001. ***C***, Primary cortical neurons were exposed to monomeric α-synuclein or α-synuclein fibrils at DIV7 from wild-type, tau heterozygous mice, or tau knock-out mice. Three days later or eleven days later, neurons were fixed and labeled with antibodies to presynaptic vGLUT1, or postsynaptic Homer. Pearson’s coefficient measuring the percent overlap of vGLUT1 over Homer was quantified using Fiji. For DIV10 *F*_(2,263)_ = 1.36, *p* = 0.26. For DIV18 Two way ANOVA interaction *F*_(2,237)_ = 6.546. Two-way ANOVA. Interaction: *F*_(2,237)_ = 6.596, *p* = 0.0012; treatment: *F*_(1,237)_ = 16.61, *p* < 0.0001; genotype: *F*_(2,237)_ = 44.0, p < 0.0001. Scale bar: 50 μm. ***D***, Representative images of labeled with antibodies to presynaptic vGLUT1 (magenta in merged image) or postsynaptic Homer (blue in merged image). Images were captured using confocal microscopy. Scale bar: 25 μm. Extended Data Figure 1-1 shows data from an internalization assay of Alexa Fluor 488-labeled fibrils demonstrated no difference in uptake of fibrils among genotypes. Extended Data Figure 1-2 shows data from a DQ-BSA assay demonstrating that neurons from tau heterozygous mice exposed to fibrils have increased lysosome activity compared neurons exposed to α-synuclein monomer.

PLA localizes protein-protein interactions (Söderberg et al., 2008; Pelayo et al., 2011) via oligonucleotides bound to secondary antibodies. α-Synuclein–tau interactions were examined in mature (14 DIV) primary cortical neuronal cultures from tau wild-type, heterozygous, and knock-out mice. Antibodies to the C terminus of α-synuclein and the microtubule binding domain of tau were used because these two domains interact *in vitro* (Dasari et al., 2019). In the absence of tau, there was no fluorescence, indicating that the PLA secondary antibodies and oligonucleotides do not nonspecifically bind to the neurons and produce fluorescence. Conversely, tau wild-type and heterozygous cultures showed high levels of fluorescence, indicating association of tau and α-synuclein (Fig. 1*A*). Primary neurons from tau heterozygous mice showed significantly less (∼30%) fluorescence than those from wild-type animals, indicating that a reduction in tau decreases the pool of tau interacting with α-synuclein.

10.1523/ENEURO.0458-20.2021.f1-1 Extended Data Figure 1-1 Internalization assay was completed as described previously (Karpowicz et al., 2017; Froula et al., 2019). Primary cortical neurons were cultured on poly-d-lysine coated MatTek dishes at a density of 5 × 10^5^ cells per plate as described above. At 7 DIV, sonicated fibrils were diluted in cold imaging media (136 mm NaCl, 2.5 mm KCl, 2 mm CaCl_2_, 1.3 mm MgCl_2_, 10 mm glucose, and 10 mm 4-(2-hydroxyethyl)-1-piperazineethanesulfonic acid) for a final concentration of 1 μg/ml and added to cultures. Plates were then incubated on ice for 30 min to allow the fibrils to bind to the plasma membrane. The cells were then incubated at 37°C for an additional 30 min to allow internalization. Images were captured using a Zeiss Axio Observer Z1 with Colibri LED illumination. Fluorescence of extracellular tagged fibrils was quenched with addition of 1 mg/ml (1 mm) trypan blue in PBS. Images were captured at an excitation of 470 nm for internalized Alexa Fluor 488-labeled fibrils and trypan blue labeled neurites at 560 nm. The images were analyzed using ImageJ by manually defining a minimum background threshold for the images After the threshold had been set for each group, the integrated density for each image was generated. Results of two independent experiments, WT (*N* = 2 independent mice), tau heterozygous (*N* = 2 independent mice), tau knock-out (*N* = 3 independent mice). Kruskal–Wallis test, *H* = 0.6935, *p* = 0.7070. Download Figure 1-1, TIF file.

**Figure 2.**
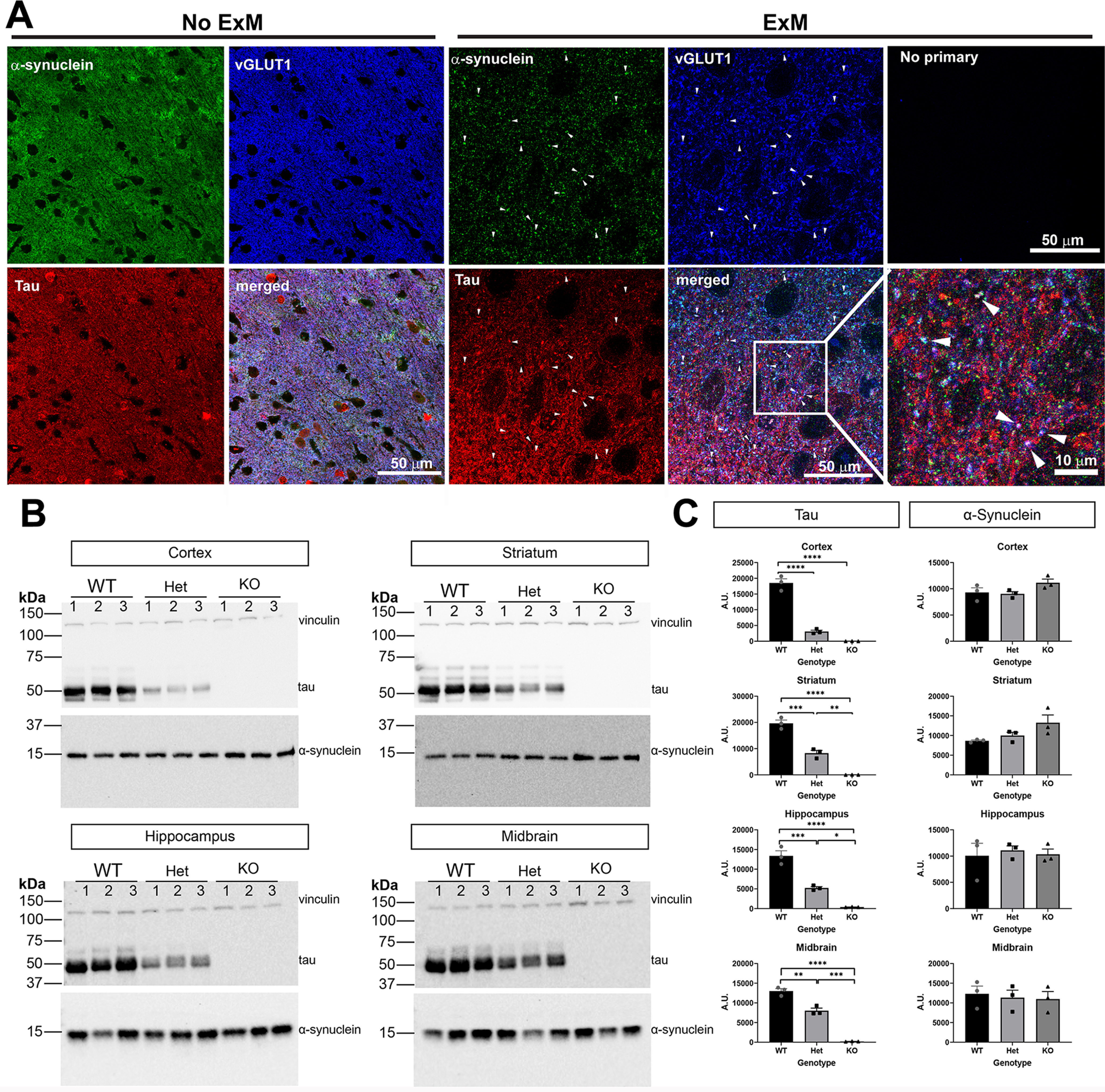
***A***, Cortical mouse section with immunofluorescence using antibodies to tau, α-synuclein, and vGLUT1. Images were scanned using confocal microscopy. Scale bar: 50 μm. ***B***, Expansion microscopy (2× expansion) was performed along with antibodies as in ***A***. Images capture using confocal microscopy. Arrowheads point to puncta in which tau, α-synuclein, and vGLUT1 overlap. The top right panel shows a confocal image from expanded sections in which no primary antibodies were included but all three secondary antibodies were included. The bottom right panel shows a higher magnification image from the panel to the left. Scale bar: 10 or 50 μm as indicated. ***C***, Immunoblots from cortex, striatum, hippocampus homogenates of three-month-old tau wild-type, heterozygous, and knock-out mice. Vinculin was used as a loading control. ***D***, Quantification of immunoblots was performed using Fiji. One-way ANOVA. Cortex (tau *F*_(2,6)_ = 151.0, *p* < 0.0001; synuclein *F*_(2,6)_ = 2.703, *p* = 0.1455). Striatum (tau *F*_(2,6)_ = 110.5, *p* < 0.0001; synuclein *F*_(2,6)_ = 3.611, *p* = 0.0934). Hippocampus (tau *F*_(2,6)_ = 73.06, *p* < 0.0001; synuclein *F*_(2,6)_ = 0.112, *p* = 0.8958). Midbrain (tau *F*_(2,6)_ = 152.1, *p* < 0.0001; synuclein *F*_(2,6)_ = 0.1251, *p* = 0.8846); *p < 0.05, **p < 0.01, ***p < 0.005, ****p < 0.0001. *N* = 3 independent mice.

10.1523/ENEURO.0458-20.2021.f1-2 Extended Data Figure 1-2 On DIV7, neurons were treated with α-synuclein fibrils or monomer (1 μg/ml). On DIV14, neurons were treated with DQ-BSA Red (Invitrogen) substrate (images are pseudo-colored green for easier visualization). DQ-BSA Red was solubilized in PBS to a stock concentration of 1 mg/ml and sonicated with a probe tip sonicator for 5 s with a 1-s pulse at 20% amplitude, followed by syringe filtering with a 0.22-μm membrane. Neurons were incubated with 25 μm DQ-BSA Red solution for 2 h at 37°C. Neurons were then fixed with 4% PFA and incubated in Hoechst before mounting the coverslips with Prolong Gold (Invitrogen). For each treatment and genotype, 15–30 random fields were captured using a Zeiss widefield microscope. Following imaging, ImageJ analysis software was used to quantify and analyze DQ-BSA substrate data. After manually drawing the neuronal region of interest containing bright fluorophores, the % area occupied by the puncta was determined using ImageJ. WT (*N* = 2 independent mice), tau heterozygous (*N* = 2 independent mice), tau knock-out (*N* = 3 independent mice). Data were analyzed by two-way ANOVA. Interaction: *F*_(2,268)_ = 7.2, *p* = 0.0009; treatment: *F*_(1,268)_ = 22.1, *p* < 0.0001; genotype: *F*_(2,268)_ = 1.678, *p* = 0.1886; ****p < 0.0001. Scale bar: 50 μm Download Figure 1-2, TIF file.

### Reducing tau does not prevent α-synuclein inclusion formation

To examine the impact of reducing tau on fibril-induced α-synuclein inclusion formation, fibrils generated from recombinant α-synuclein were added to neurons from wild-type, tau heterozygous, or tau knock-out mice. The formation of inclusions, identified using an antibody to p-α-synuclein, was quantified using immunofluorescence and confocal microscopy. The abundance of α-synuclein inclusions was not reduced in neurons from either tau heterozygous mice or tau knock-out mice compared with wild-type mice (Fig. 1*B*). Overall, reducing tau did not inhibit fibril-induced α-synuclein inclusion formation in primary cortical neurons.

α-Synuclein expression and fibril-induced α-synuclein inclusion formation depends on synapse formation (Murphy et al., 2000; Volpicelli-Daley et al., 2011), which may be impaired by reducing tau (Dawson et al., 2001). Thus, we measured the colocalization of presynaptic vGLUT1 and postsynaptic Homer to determine whether reducing tau inhibits synapse formation. We also examined whether adding fibrils reduced synapses, by adding α-synuclein fibrils at DIV7 and measuring overlap of vGLUT1 and Homer (Fig. 1*C*,*D*). Synapses begin to mature by DIV10 (Fletcher et al., 1994). At this time point, there were no significant differences in the overlap of vGLUT1 and Homer in neurons with reduced tau, or neurons 3 d following exposure to fibrils. At DIV18, however, tau heterozygous neurons (but not tau knock-out neurons) showed increased overlap of vGLUT1 and Homer (Fig. 1*C*,*D*). vGLUT1 immunofluorescence in particular appeared brighter with larger puncta, suggesting that reducing tau by 50% enhances synapse formation. Overall, the data showed that reducing tau does not inhibit synapse formation. In wild-type neurons, addition of fibrils also increased overlap of vGLUT1 and Homer with vGLUT1 puncta appearing brighter and larger, consistent with findings of increased volume of presynaptic terminals in the cortex and striatum in DLB cases (Colom-Cadena et al., 2017). We also determined whether tau reduction impairs fibril internalization. Neither neurons from tau heterozygous mice nor tau knock-out mice showed alterations in the internalization of Alexa Fluor 488-labeled α-synuclein fibrils compared with neurons from wild-type controls (Extended Data Fig. 1-1) (Karpowicz et al., 2017). Because turnover of α-synuclein could also impact aggregation, lysosome activity was also measured using a DQ-BSA, which fluoresces when cleaved by lysosomal proteases. Addition of fibrils significantly increased the abundance of active lysosomes in the tau heterozygous primary neurons at 7 d after adding fibrils, compared with neurons exposed to monomer (Extended Data Fig. 1-2).

### Tau partially overlaps with α-synuclein at excitatory synapses

α-Synuclein concentrates at the presynaptic terminal, particularly in excitatory neurons in the cortex (Taguchi et al., 2016). Tau localizes to the axon and plays a role in synaptic vesicle endo/exocytosis at the presynaptic terminal (Zhou et al., 2017; McInnes et al., 2018). Tau expression is also enriched in excitatory neurons in the cortex (Fu et al., 2019). We first examined localization of α-synuclein and tau in cortical sections of wild-type mice. Using indirect immunofluorescence and traditional confocal microscopy, α-synuclein, tau, and vGLUT1, a presynaptic excitatory neuron marker, appeared diffuse throughout the neuropil of the cortex (Fig. 2*A*). To increase our resolution, we used expansion microscopy, which utilizes a polymer matrix to mechanically expand tissue evenly in 3 dimensions (Chen et al., 2015; Tillberg et al., 2016; Wassie et al., 2019). Using this technique along with traditional confocal microscopy, we expanded our cortical tissue sections. Distinct puncta corresponding to α-synuclein and excitatory vGLUT1 presynaptic puncta were visible. A portion of tau immunofluorescence overlapped with α-synuclein and vGLUT1, indicating that tau can localize to the presynaptic terminal along with α-synuclein (Fig. 2*A*, bottom panels).

### Tau reduction does not affect α-synuclein expression

We determined whether tau reduction affects endogenous α-synuclein levels in the brain, which could influence the extent of α-synuclein aggregation. Homogenates of hippocampus, cortex, striatum, and midbrain were analyzed for levels of tau and α-synuclein by immunoblot (Fig. 2*B*). As expected, tau heterozygous mice showed an ∼50% reduction of tau protein relative to wild-type littermates in all brain regions. Tau knock-out mice showed no tau expression. In all four brain regions analyzed, α-synuclein levels were not significantly different regardless of tau expression (Fig. 2*C*). Although there appeared to be a trend toward an increase in α-synuclein expression in the cortex and striatum, our data were not powered to detect a significant difference.

### Tau reduction does not prevent inclusion formation at six months after injection of α-synuclein fibrils

**Figure 3.**
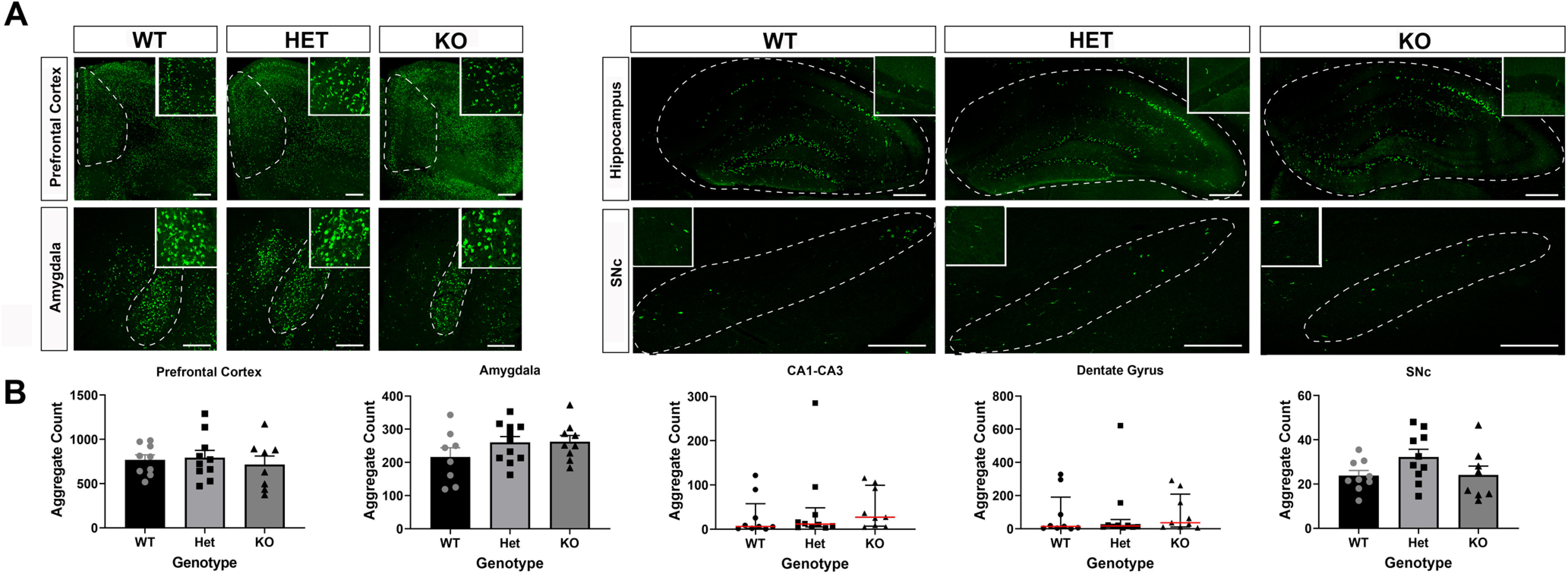
***A***, Six months after bilateral striatal injection of α-synuclein fibrils, mice were perfused and immunofluorescence to p-α-synuclein was performed. Images were captured using confocal microscopy and p-α-inclusions were quantified using Fiji. Higher magnification inserts are provided for visualization of inclusions. ***B***, Quantification of PFC (left), basolateral amygdala (center left), dentate gyrus of the hippocampus (center), CA1–CA3 of the hippocampus (center right) and SNc (right). PFC: one-way ANOVA, *F*_(2,24)_ = 0.2523, *p* = 0.7791. Basolateral amygdala: one-way ANOVA, *F*_(2,25)_ = 1.368, *p* = 0.2731. Dentate gyrus: Kruskal–Wallis test, *H* = 1.161, *p* = 0.3223. CA1–CA3: Kruskal–Wallis test, *H* = 2.265, *p* = 0.5597. SNc: one-way ANOVA, *F*_(2,24)_ = 2.119, *p* = 0.1421. *N* = 8–10/group. Scale bars: 250 μm. Extended Data Figure 3-1 shows data from unbiasaed stereology counts of α-synuclein aggregates in the amygdala confirming no differences among fibril-injected wild-type, tau heterozygous, and tau knock-out mice.

To evaluate the role of tau on Lewy-like aggregation, wild-type, heterozygous, and knock-out tau mice received bilateral injections of α-synuclein fibrils or monomer at three to four months of age. Injection of α-synuclein fibrils into the striatum induces Lewy-like aggregates in multiple brain regions important for LBDs (Luk et al., 2012; Froula et al., 2019; Stoyka et al., 2020), including the PFC, basolateral amygdala, striatum, and SNc. Monomeric α-synuclein was injected as a control, which does not induce formation of α-synuclein inclusions (Volpicelli-Daley et al., 2011; Froula et al., 2019). At six to seven months postinjection, mice were killed, and immunofluorescence was performed using an antibody to α-synuclein phosphorylated at Ser129 (p-a-syn) to identify inclusions (Fig. 3). In both humans and in murine models, α-synuclein is hyperphosphorylated in aggregates (Fujiwara et al., 2002), this antibody recognizes Lewy aggregates in PD and DLB brains (Fujiwara et al., 2002). The PFC and basolateral amygdala contained heavy burdens of Lewy-like aggregates, consistent with previous observations (Luk et al., 2012; Froula et al., 2019; Sorrentino et al., 2019; Stoyka et al., 2020). The burden in the hippocampus was variable regardless of genotype. In general, the dentate gyrus had a higher burden than CA1–CA3, but the number of Lewy-like aggregates varied from few aggregates to hundreds. Compared with the other brain regions, the SNc had few aggregates, consistent with previous studies showing an increase in fibril induced α-synuclein aggregates at three months postinjection followed by decreases at six months postinjection, likely because of concomitant death of aggregate-containing neurons (Abdelmotilib et al., 2017; Patterson et al., 2019). Indeed, tracking neurons *in vivo* has shown death following fibril-induced aggregate formation (Osterberg et al., 2015). Quantification of aggregates revealed that the abundance of α-synuclein inclusions was not significantly different in the cortex, amygdala, hippocampus, or SNc among wild-type, tau heterozygous, or tau knock-out mice (Fig. 3*B*). Unbiased stereology was also used to quantify aggregates in the basolateral amygdala, and confirmed that tau expression does not impact the abundance of fibril-induced inclusions (Extended Data Fig. 3-1).

10.1523/ENEURO.0458-20.2021.f3-1 Extended Data Figure 3-1 Unbiased stereology was performed to count p-α-synuclein aggregates in the basolateral amygdala in mice six months after fibril injections. Unbiased stereological analyses were conducted on a fluorescence microscope using an optical fractionator probe (Stereo Investigator software, Stereology Resource Center) on sections stained for phosphorylated synuclein. For stereology of the basolateral amygdala, a 4× objective was used to identify the borders, then a 40 × 0.75 NA objective was used to count p-syn. Sections covered the entire BLA amygdala and were equally spaced 200 μm apart. A total of four to six sections per animal were quantified. Serial sectioning was used to identify sections between bregma coordinates –2.18 to –0.82 mm. The optical dissector height was 22 μm, and the distance between the counting frame was 50 × 50 μm. The grid size was 150 × 150 μm. The counting variability was measured with the Schmitz–Hof CE and was 0.064. One-way ANOVA. Interaction: *F*_(2,21)_ = 0.4911, *p* = 0.618; treatment. *N* = 8/group. Download Figure 3-1, TIF file.

### Tau reduction does not prevent loss of TH-positive neurons in the SNc at six months after injection of α-synuclein fibrils

To determine the effect of tau reduction on α-synuclein inclusion-induced loss of dopamine neurons in the SNc, immunohistochemistry of serial sections of the midbrain was performed using an antibody to TH, a biosynthetic enzyme for dopamine, to identify dopaminergic neurons. TH-positive neurons were quantified using unbiased stereology. At six to seven months after injection of α-synuclein fibrils, there was an ∼50% bilateral loss of tyrosine-hydroxylase positive neurons in the SNc relative to mice injected with α-synuclein monomer, consistent with previous reports in mice and rats (Luk et al., 2012; Paumier et al., 2015). There were no differences in TH-positive neuron counts within genotype in either α-synuclein monomer injected mice or α-synuclein fibril mice. 

**Figure 4.**
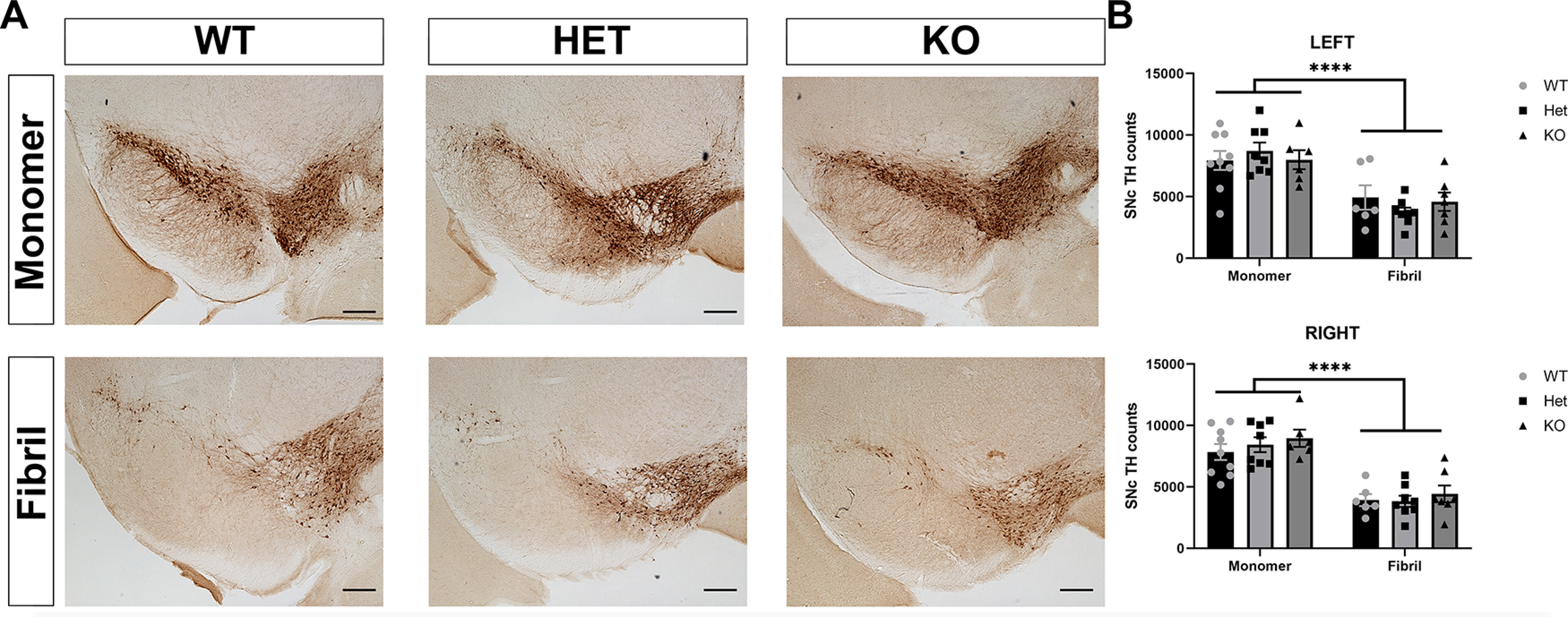
***A***, Wild-type (*N* = 9, monomer; *N* = 6, fibril), tau heterozygous (*N* = 8, monomer; *N* = 8, fibril), or tau knock-out (*N* = 6, monomer; *N* = 7, fibril) tau mice were perfused six months after injections. Immunohistochemistry was performed using an antibody to TH to identify dopamine neurons. ***B***, Unbiased stereology was performed to count TH-positive neurons in both the right and left SNc. The only significant difference was between monomer and fibril injection with no significant effect of genotype. Two-way ANOVA. Interaction: *F*_(2,38)_ = 0.5941, *p* = 0.5571; treatment: *F*_(1,38)_ = 61.12, *p* < 0.0001; genotype: *F*_(2,38)_ = 0.1630, *p* = 0.8502. *N* = 6–8/group; ****p < 0.0001. Scale bar: 200 μm.

### Behavior phenotypes depend on tau expression, both dependent and independent of α-synuclein inclusion formation

**Figure 5.**
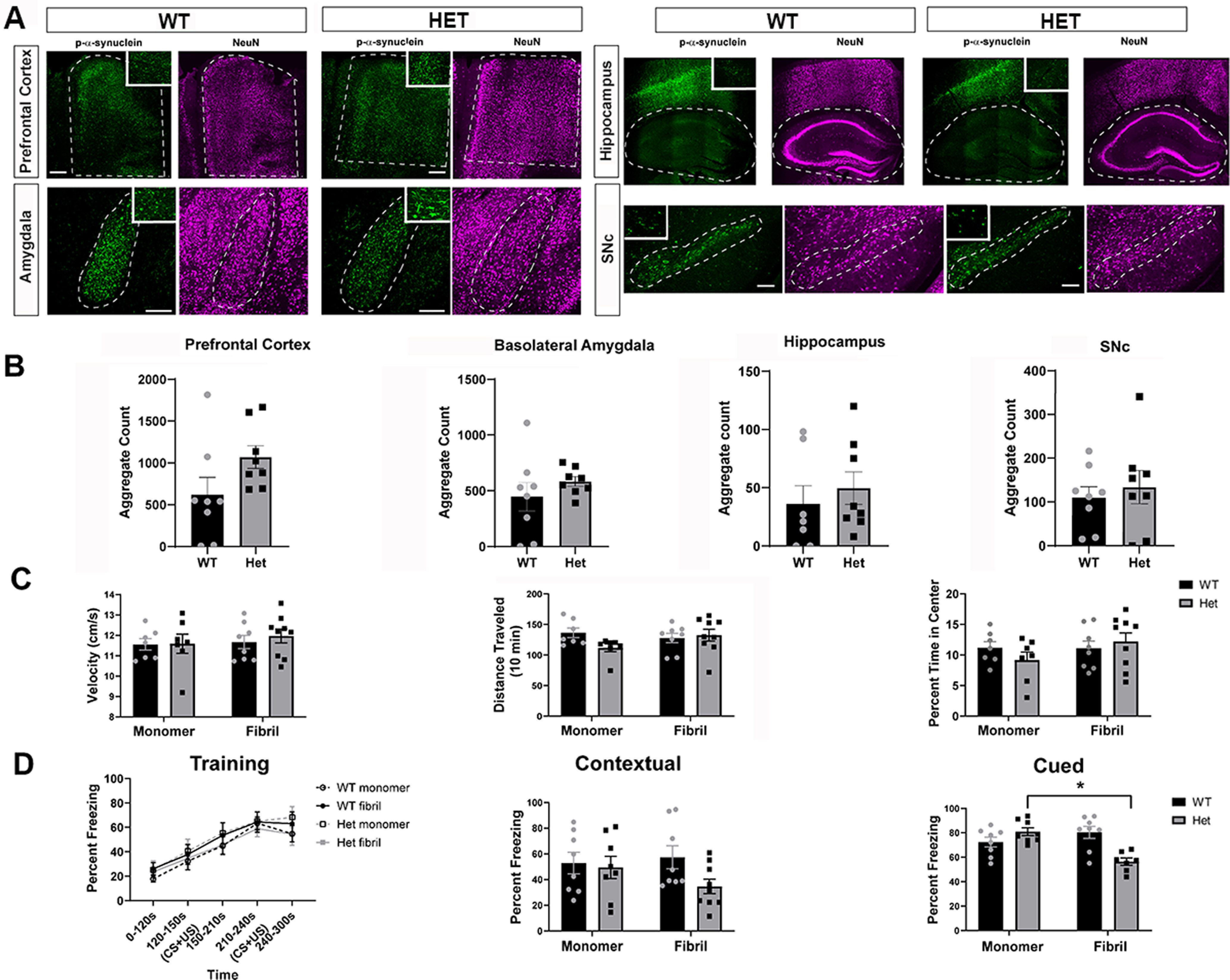
Mice underwent behavior testing five to six months after injection of monomer or fibrils. ***A***, Analyses of open field test: velocity, total distance traveled (cm), and percentage of time in center. Two-way ANOVA. Velocity, one outlier removed; interaction: *F*_(2,48)_ = 2.700, *p* = 0.0774; treatment: *F*_(1,48)_ = 3.248, *p* = 0.0778; genotype: *F*_(2,48)_ = 2.805, *p* = 0.0705. Distance traveled, interaction: *F*_(2,49)_ = 4.9, *p* = 0.01; treatment: *F*_(1,49)_ = 0.79, *p* = 0.38; genotype: *F*_(2,49)_ = 3.7, *p* = 0.03. Percent time in center, interaction: *F*_(2,49)_ = 0.7311, *p* = 0.4866; treatment: *F*_(1,49)_ = 0.1434, *p* = 0.7066; genotype: *F*_(2,49)_ = 0.7588, *p* = 0.4736. *p < 0.05. ***B***, Training in fear conditioning paradigm and percentage time freezing in contextual and cued tests. Training: three-way ANOVA. Three-way interaction: *F*_(8,170)_ = 0.64, *p* = 0.7449; genotype × treatment: *F*_(2,170)_ = 0.9, *p* = 0.4075; genotype × time: *F*_(8,170)_ = 0.54, *p* = 0.8246; treatment × time: *F*_(4,170)_ = 0.16, *p* = 0.9583; treatment: *F*_(1,170)_ = 0.76, *p* = 0.385; genotype: *F*_(2,170)_ = 7.44, *p* = 0.0008; time: *F*_(4,170)_ = 21.57, *p* < 0.0001. Contextual: two-way ANOVA. Interaction: *F*_(2,46)_ = 0.7843, *p* = 0.4624; treatment: *F*_(1,46)_ = 0.003584, *p* = 0.9525; genotype: *F*_(2,46)_ = 1.826, *p* = 0.1725. Cued: two-way ANOVA. Interaction: *F*_(2,49)_ = 4.003, *p* = 0.0245; treatment: *F*_(1,49)_ = 1.171, *p* = 0.2844; genotype: *F*_(2,49)_ = 3.907, *p* = 0.0266. **p < 0.01.

To determine whether Lewy-like inclusions correlated with behavioral phenotypes, we performed behavioral assays six months after injection. In the open field, tau heterozygous mice injected with fibrils showed lower total ambulatory distance than wild-type mice injected with fibrils (Fig. 5*A*). There were no significant differences among groups with respect to average velocity, or percent time in center, a measure of anxiety.

All mice successfully learned a fear conditioning paradigm (Fig. 5*B*). No group was significantly different from tau wild-type monomer mice at any time point during training; a three-way ANOVA revealed significant differences for genotype and time, but not treatment. In contextual fear conditioning, mice were re-exposed to the physical environment associated with the foot shock and freezing time was measured to determine memory of the adverse event. All groups of mice showed equivalent freezing behavior during contextual fear conditioning when compared with wild-type monomer mice. In cued fear conditioning, mice were placed in a novel environment and re-exposed to the auditory tone previously associated with the adverse stimulus. Statistical analysis using two-way ANOVA and *post hoc* tests revealed a significant difference between monomer injected wild-type and monomer injected knock-out mice (Fig. 5*B*), tau knock-out monomer-injected mice froze significantly less than wild-type monomer-injected mice.

### Tau reduction does not prevent inclusion formation at 1.5 months after injection of α-synuclein fibrils

**Figure 6.**
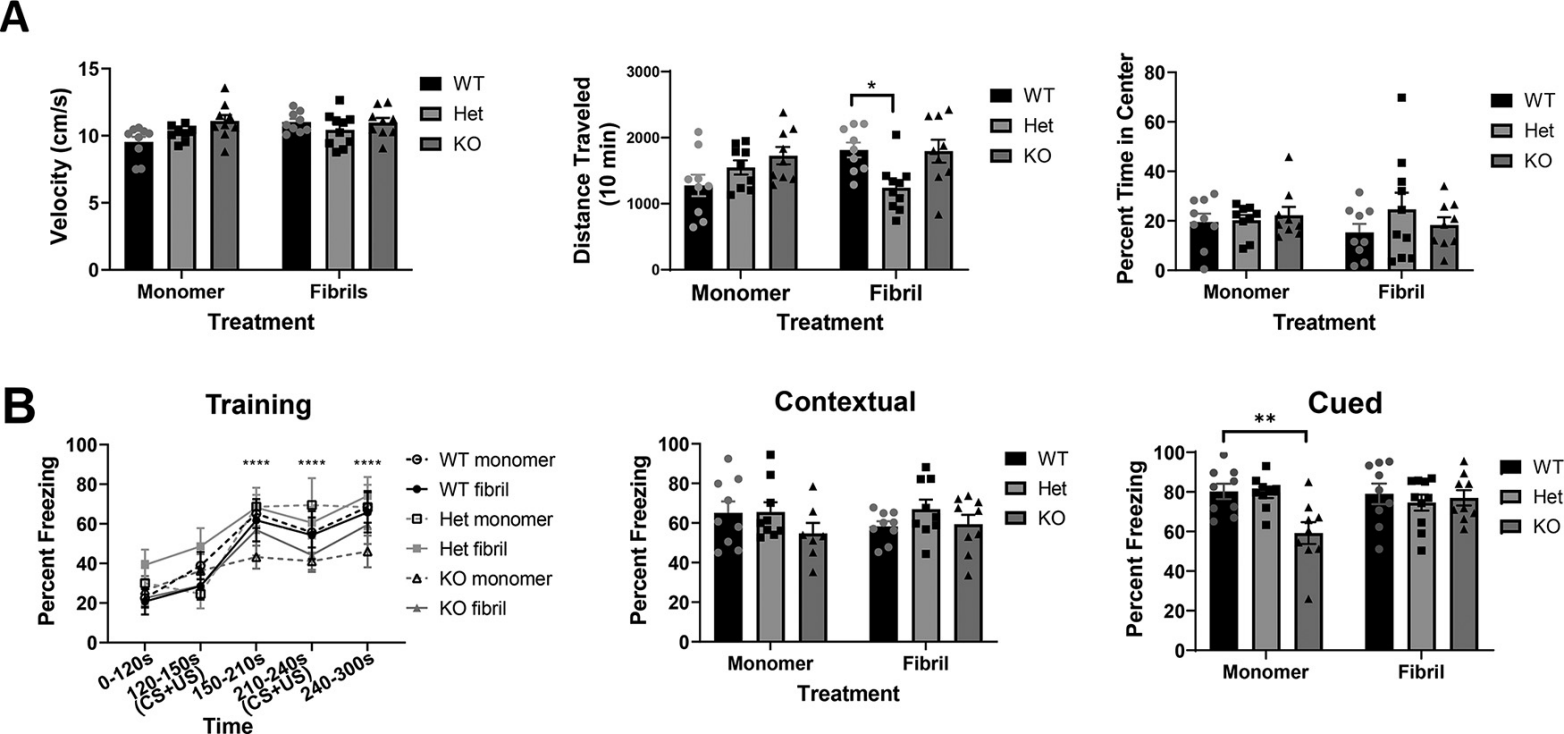
***A***, One and a half months after bilateral striatal injection of α-synuclein fibrils, mice were perfused and immunofluorescence to p-α-synuclein was performed. Images were captured using confocal microscopy and p-α-inclusions were quantified using Fiji. Higher magnification insets in each panel show inclusions. ***B***, Quantification of PFC (left), basolateral amygdala (center left), hippocampus (center right) and SNc (right). PFC: independent *t* test, *t*_(14)_ = 1.8, *p* = 0.09. Basolateral amygdala: independent *t* test, *t*_(14)_ = 1.02, *p* = 0.32. Hippocampus, *t*_(13)_ = 0.65, *p* = 0.52. SNc *t*_(14)_ = 0.53, *p* = 0.6. *N* = 8–10/group. Scale bars: 250 μm. ***C***, Analyses of open field test: velocity, total distance traveled (cm), and percentage of time in center. Two-way ANOVA. Velocity, interaction: *F*_(1,27)_ = 3.1, *p* = 0.09; treatment: *F*_(1,27)_ = 0.5, *p* = 0.5; genotype: *F*_(1,27)_ = 1.4, *p* = 0.24. Distance traveled, interaction: *F*_(1,27)_ = 0.13, *p* = 0.71; treatment: *F*_(1,27)_ = 0.47, *p* = 0.49; genotype: *F*_(1,27)_ = 0.2, *p* = 0.65. Percent time in center, interaction: *F*_(1,27)_ = 1.5, *p* = 0.23; treatment: *F*_(1,27)_ = 1.3, *p* = 0.26; genotype: *F*_(1,2)_ = 0.12, *p* = 0.72. ***D***, Training in fear conditioning paradigm and percentage time freezing in contextual and cued tests. Training: three-way ANOVA. Three-way interaction: *F*_(16,120)_ = 0.8, *p* = 0.67; genotype × treatment: *F*_(4,30)_ = 0.5, *p* = 0.71; time: *F*_(2,88)_ = 42.8, p < 0.0001. Contextual: two-way ANOVA. Interaction: *F*_(1,29)_ = 1.4, *p* = 0.23; treatment: *F*_(1,27)_ = 0.43, *p* = 0.51; genotype: *F*_(1,29)_ = 2.8, *p* = 0.11. Cued: two-way ANOVA. Interaction: *F*_(1,27)_ = 17.52, *p* = 0.0003; treatment: *F*_(1,27)_ = 4.5, *p* = 0.04; genotype: *F*_(1,27)_ = 3.9, *p* = 0.05. *p < 0.05.

It was possible that six months after fibril injections, potential differences in the abundance of α-synuclein inclusions are minimal at this late time point. However, analyzing an earlier time point after fibril injections could reveal differences among phenotypes. We therefore performed bilateral injections of α-synuclein fibrils into the striatum and analyzed p-α-synuclein inclusions 1.5 months later. We focused on wild-type and tau heterozygous mice because any therapeutic approach would reduce, but not completely remove, tau. The PFC, basolateral amygdala, and SNc showed robust formation of inclusions 1.5 months after striatal fibril injections (Fig. 6*A*). At this time point, α-synuclein inclusions in the hippocampus were scant and restricted to small aggregates in the dentate gyrus. Quantitation of the inclusions revealed no significant differences between wild-type and tau heterozygous mice in any brain region (Fig. 6*B*).

We performed behavioral assays 1.5 months after injection of fibrils. There were no significant differences between wild-type and tau heterozygous mice injected with monomer or fibrils in open field analyses including velocity, ambulatory distance and percent time in center (Fig. 6*C*). All mice successfully learned a fear conditioning paradigm (Fig. 6*D*). No group was significantly different from tau wild-type monomer mice at any time point during training; a three-way ANOVA revealed significant differences for time, but not treatment or genotype. All groups of mice showed equivalent freezing behavior during contextual fear conditioning when compared with wild-type monomer mice. In cued fear conditioning, the tau heterozygous fibril-injected mice froze less than wild-type fibril-injected mice.

## Discussion

Here, we showed using PLA and expansion microscopy that tau and α-synuclein both localize in presynaptic terminals, consistent with previous findings from synaptosome preparations from human brains, and consistent with *in vitro* studies showing that both proteins interact (Arima et al., 1999; Giasson et al., 2003; Galpern and Lang, 2006; Moussaud et al., 2014). However, reduction or complete absence of tau did not prevent fibril-induced α-synuclein inclusion formation in primary hippocampal neurons or *in vivo*, in the cortex, amygdala, hippocampus, or SNc at either early or late time points after fibril injections, consistent with a previous report (Bassil et al., 2021). Additionally, although we saw a robust, ∼50% loss of dopaminergic neurons in the SNc induced by α-synuclein fibrils, reduction of tau did not prevent this loss. Lastly, reducing tau did not have any major impact behavioral phenotypes in mice with fibril-induced α-synuclein inclusions.

Reducing tau in, human A53T-α-synuclein α-synuclein transgenic mice rescued cognitive behavioral phenotypes (Singh et al., 2019). This model relies on increased expression of mutant, human α-synuclein. Expression of this rare α-synuclein mutation forms Lewy-like aggregates in the cerebellum and pons but does not form insoluble aggregates in brain regions such as the cortex, amygdala or hippocampus. It is possible that the phenotypes such as impaired fear conditioning and synaptic defects are caused by increased expression of human A53T-α-synuclein or small oligomers. While α-synuclein gene multiplication and polymorphisms can slightly increase levels of α-synuclein and cause PD (Singleton et al., 2003), it is currently unclear whether abnormal aggregation is a gain of toxic function or a loss of function. Our lab previously showed that early formation of seeded α-synuclein inclusions causes an initial increase in synaptic vesicle release (Froula et al., 2018). Normally, α-synuclein acts as a brake on release of synaptic vesicles (Sun et al., 2019) and thus, our data point to aggregation as a loss of α-synuclein function. Also, our data are consistent with other studies showing that reduction of tau in mice expressing human wild-type α-synuclein was not effective in preventing motor defects (Morris et al., 2011). Overall, our results indicate that endogenous tau does not play a role in seeded α-synuclein inclusion formation. However, because tau pathology is present in a subset of patients with PD, it is possible that abnormal tau assemblies are playing a role in PD phenotypes and that reduction of oligomers may be protective (Gerson et al., 2018).

It is essential to consider the limitations of the current study and the degree to which it recapitulates human LBDs. First, we cannot exclude the possibility that embryonic knock-out or partial reduction of tau induces a compensatory mechanism by which a potential effect of tau is negated. To counter this, an inducible tau knock-out line or tau antisense oligonucleotides could be beneficial. Additionally, any extrapolation to human disease is limited in the etiology of sporadic LBD, as our model “skips” the initial insult and multifactorial causes of the disease via intracerebral injection to induce Lewy-like pathology. Our data suggest that tau does not play a significant role after the initial insult, but it remains possible that tau may affect the initial misfolding of α-synuclein.

Our behavioral data showed that homozygous tau knock-out caused defects in cued fear conditioning in aged (nine month) mice which has been demonstrated previously (Ikegami et al., 2000; Ahmed et al., 2014; Goncalves et al., 2020) and may be related to their tendency toward hyperactivity, since other learning and memory tests are not impaired. Tau heterozygous (but not tau knock-out) mice injected with fibrils showed defects compared with control mice in cued fear conditioning at an early time point following injections. Our findings that tau reduction does not rescue behavioral phenotypes support previous studies showing that tau reduction does not improve behavioral phenotypes in 6-hydroxydopamine or MPTP lesion models, or in α-synuclein transgenic mice (Morris et al., 2011; Gratuze et al., 2019). However, antibodies directed to tau oligomers do show protection in α-synuclein models and it therefore remains possible that tau immunotherapy may be protective in synucleinopathies with co-existing pathologic aggregates of tau (Gerson et al., 2018).

Tau reduction is an attractive avenue for potential therapeutics in multiple neurodegenerative diseases because of its role in aggregation, potential as a biomarker, and avenue for immunotherapy (Gerson et al., 2018). Indeed, studies using transgenic lines for AD and tauopathies have shown that approach to be promising (Roberson et al., 2007; DeVos et al., 2018). However, the results of this study suggest the role of tau in LBDs may not be as forthright as in AD, and the two spectra of diseases may have distinct pathogenesis as far as tau is concerned. Data here suggests that any role of endogenous, physiologic tau (at least in a murine model) is upstream to the fibrillization of α-synuclein pathology. Additional studies are needed to further explore the therapeutic benefit of tau reduction in LBDs, but these results suggest that Lewy-like pathology is independent of tau.

## Synthesis

Reviewing Editor: Mikko Airavaara, University of Helsinki Decisions are customarily a result of the Reviewing Editor and the peer reviewers coming together and discussing their recommendations until a consensus is reached. When revisions are invited, a fact-based synthesis statement explaining their decision and outlining what is needed to prepare a revision will be listed below. The following reviewer(s) agreed to reveal their identity: Caryl Sortwell.

The manuscript “Templated α-synuclein inclusion formation is independent of endogenous tau” is very well prepared, clear and the methodology is proper for given questions and meticulously described. Tau heterozygous and tau KO mice are used to manipulate tau levels. Overall the study is very well conducted with the exception of the few specific points raised below. The finding is of negative nature (lack of impact of Tau on aSyn aggregation) but important in light of other findings suggesting interaction of this proteins. The novelty of the finding is diminished by recent publication [1]. However we think that replicating such studies is very important especially that additional in vitro methods were utilized. Moreover, the important novel finding of this study, especially from clinical perspective, is that knocking out or reducing Tau does not protect for aSyn PFF induced degeneration of dopaminergic neurons.

The following suggestions are made to improve the manuscript.

1. Bassil, F.; Meymand, E.S.; Brown, H.J.; Xu, H.; Cox, T.O.; Pattabhiraman, S.; Maghames, C.M.; Wu, Q.; Zhang, B.; Trojanowski, J.Q.; et al. α-Synuclein modulates tau spreading in mouse brains. J. Exp. Med. 2021, 218, doi:10.1084/jem.20192193.

1) Overall the analysis of the whether alpha-synuclein inclusion formation is impacted by tau is relatively limited, analyzed solely by quantifying pSyn inclusion load. This conclusion would be strengthened by examination of additional inclusion properties, other antibodies against pathological alpha-syn or differences in Proteinase K resistance. The quantitation approach of magnitude of pSyn aggregates is also not particularly rigorous in the in vivo experiment. pSyn level in a single 40um section within each brain structure is quantified but this does not take into account variability of seeding across a particular structure. TH neurons were quantified using stereology, why not a similar approach to quantifying pSyn?

2)

Authors should to mention either in introduction or discussion publication

Bassil, F.; Meymand, E.S.; Brown, H.J.; Xu, H.; Cox, T.O.; Pattabhiraman, S.; Maghames, C.M.; Wu, Q.; Zhang, B.; Trojanowski, J.Q.; et al. α-Synuclein modulates tau spreading in mouse brains. J. Exp. Med. 2021, 218, doi:10.1084/jem.20192193.

3) Results show that reductions in tau levels do not influence the formation of phosphorylated alpha-synuclein inclusions in vitro or in vivo - however this is misstated in the abstract, “However, tau heterozygous and tau knockout mice did show a reduction in fibril induced α-synuclein inclusions formation in primary cortical neurons or after intrastriatal injections of fibrils at 1.5 month or 6 months later.” This needs to be corrected.

4) In the introduction: “The effects of tau reduction in synucleinopathy models...” probably should state in something like “neurotoxic or synculeinopatic PD models” as authors in previous sentence mention MPTP and 6OHDA models.

5) In Materials and Methods Animals: the authors sufficiently described transgenic animals (which is not always done) but they should also give reference allowing the interested reader to find details about these animals

6) In Materials and Methods Preparation of Fibrils: this section is very well described. The authors could also clarify how were PFFs kept between sonication and injections (they only state that during injections, they were kept at RT, but do not say if sonication was done each day, or maybe batches were stored frozen).

7) In Materials and Methods Preparation of Fibrils: Dynamic light scattering is very useful in checking between batch variation of sonicated fibrils, but as far as we know absolute quantification of the size of elongated particles is troublesome with this method (it work best for round particles). Are authors sure they can state the 60nm size of fibrils from DLS measurement? If this is approximate, please mention it.

8) In Materials and Methods: Authors should provide specific RRIDs of all antibodies.

9) In Materials and Methods Behavior testing: authors mention “tube test,” but it is not later reported.

10) In Materials and Methods Behavior testing: authors write about main experiments done after 6 months, but should also mention here experiment done 1.5 months after PFFs injections.

11) In Materials and Methods Behavior testing: what was the open field container size? What was illumantion (lux)? This is important if open field is used as anxiety measure.

12) There are two results sections with the same title: “Tau reduction does not prevent inclusion formation or neurodegeneration at 6 months after injection of α-synuclein fibrils.” This needs to be corrected to reflect the 1.5 month time point instead. It is suggested that the results of inclusion formation in vivo at both time points be combined in the results section followed by the behavioral data from both time points, with the earlier time point presented first. In addition, a description of the 1.5 month euthanasia time point does not appear in the methods along with number of mice in this portion of the study.

13) In Fig1D and other. The authors quantify together both somal and neuritic aggregates. For in vivo experiments it’s difficult to distinguish them unequivocally. But for in vitro experiments it’s possible to separately count the number of neurons (neuN positive cells) harboring aggregates in cell soma. This would be interesting considering Tau axonal localization interaction occur in neurites. Could the authors provide such quantification?

14) Figure 1: The descriptor of red to indicate the vGlut in B should be modified as perhaps a better descriptor would be pink as it looks more pink in the version I’m reviewing. Conceptually, it might make more sense to present the data for the

lack of difference in inclusion formation between the three tau genotypes (D) before discussing the Homer vGLUT1 colocalization data. Also, the spacing of the lower fibril panel in B could be optimized for clarity that it is part of B.

15) Figure 2: It would be helpful to see primary antibody deleted examples or blocking peptide controls in both the case of non-expanded and expanded tissue to convincingly demonstrate not background immunofluorescence. Fig 2B - insets with bigger magnification would be beneficial.In Figure 2D it looks like for both the cortex and striatum there is a trend toward an increase in a-syn expression with KO of tau, is the experiment powered appropriately to determine whether this is an actual difference?

16) Figure 3: The addition of higher magnification inserts would allow for better appreciation of the inclusions. Same suggestion for Figure 6. Also, consider y axis break for graphing the dentate and CA1-CA3 graphs.

17) Figure 4: Please present actual TH positive counts either in the text or graphed instead of percentages in the figure. Are there any differences in TH neuron number due to the genotype? The image of the tau KO monomer mouse condition looks like the SN may have fewer neurites/neurons - is this image representative? Also, Authors compare monomer to fibril injected animals. However in text they state: “α-Synuclein monomer-injected animals showed no loss of TH-positive neurons in the SNc” how this is know when there was no “sham/untreated” group and injections were bilateral?

18) The authors don’t discuss genotype×treatment interaction they mention for figure 5B. “Statistical analysis showed the interaction of genotype and treatment to be significant, as well as genotype alone, but not treatment independently (Figure 5B)

19) Second section results section, first sentence, missing a word:

"α-Synuclein expression and fibril-induced α-synuclein inclusion formation depends synapse formation (Murphy et al., 2000; Volpicelli-Daley et al., 2011).”

20) Journal guidelines should be checked for visual abstract, RRIDs and statistical table. There is no statistical methods section presented.

## Author Response

We are thankful to the reviewers for their extremely helpful suggestions that improved the manuscript. We are happy the reviewers thought that “Overall the study is very well conducted", and that the “important novel finding of this study, especially from clinical perspective, is that knocking out or reducing Tau does not protect for aSyn PFF induced degeneration of dopaminergic neurons.”

The comments (blue) are listed below and our responses follow in black.

1) Overall the analysis of the whether alpha-synuclein inclusion formation is impacted by tau is relatively limited, analyzed solely by quantifying pSyn inclusion load. This conclusion would be strengthened by examination of additional inclusion properties, other antibodies against pathological alpha-syn or differences in Proteinase K resistance. The quantitation approach of magnitude of pSyn aggregates is also not particularly rigorous in the in vivo experiment. pSyn level in a single 40um section within each brain structure is quantified but this does not take into account variability of seeding across a particular structure. TH neurons were quantified using stereology, why not a similar approach to quantifying pSyn?

We performed unbiased stereology of pSyn aggregates in the basolateral amygdala where we see abundant inclusions and a boundary can easily be drawn for stereological counts. Using this method, there are no differences in the abundance of inclusions among the genotypes, similar to our approach of counting inclusions using Fiji software. These data have been included in Extended Data Figure 3-1.

We have attempted to perform proteinase K digestion and immunohistochemistry for total alpha-synuclein in wild type, tau heterozygous and tau knockout mice. Unfortunately, we have not had success and the immunohistochemistry appears to be very strange and not at all resembling α-synuclein inclusions; it is almost as if we are bringing out blood vessel staining (see figure of immunohistochemistry in the cortex from below). We have tried different concentrations of proteinase K ranging from 0.5 to 50 μ;g/mL, we have tried using batches of proteinase K from other labs. We have tried Syn1 antibody (BDbiosciences clone 42) at concentrations ranging from 1:5000- 1:250. We have tried Abcam rabbit monoclonal EP1646Y at concentrations ranging from 1:5000-1:250. We have tried altering the salt concentration in our buffer. We have tried performing IHC with and without antigen retrieval. Our protocol is generally as follows:

•Perfuse mice with saline followed by 4% paraformaldehyde, postfix 24 hours with 4% paraformaldehyde

•Freeze brains, cut 40 μ;m sections with sliding microtome

•Rinse sections

•Incubate with 3% H2O2

•Rinse with TBS

•Incubate in 5% normal goat serum/0.1% Tx-100

•Incubate in primary overnight in TBS and 5% goat serum

•Rinse, Incubate in goat anti-species, biotinylated secondary

•Rinse, incubate in avidin biotin complex solution

•Rinse, incubate in DAB until developed

We may not be having success because we are not using paraffin embedded sections. If the reviewer strongly believes we should show proteinase K resistance, we are happy to keep trying, but any advice would be appreciated.

2)Authors should to mention either in introduction or discussion publication

Bassil, F.; Meymand, E.S.; Brown, H.J.; Xu, H.; Cox, T.O.; Pattabhiraman, S.; Maghames, C.M.; Wu, Q.; Zhang, B.; Trojanowski, J.Q.; et al. α-Synuclein modulates tau spreading in mouse brains. J. Exp. Med. 2021, 218, doi:10.1084/jem.20192193.

We have added this reference to both the introduction and discussion as follows.

Introduction:

"The role of tau reduction in PD and LBDs has been more challenging to elucidate. In mice overexpressing human α-synuclein, absence of tau rescues memory deficits as tested by Barnes maze, contextual fear conditioning, and novel object recognition (Singh et al., 2019). Antibodies to oligomeric tau reduce memory defects and Lewy-body like pathology in mice overexpressing the A53T mutant form of α-synuclein (Gerson et al., 2018). In contrast, absence of tau did not prevent templated spreading of misfolded α-synuclein, or dopamine neuron loss and associated motor deficits in toxin-induced and transgenic models of human-α-synuclein overexpression (Morris et al., 2011; Gratuze et al., 2019; Jiao et al., 2020; Bassil et al., 2021). The effect of tau reduction in synucleinopathy models, therefore, differs from AD models and further characterization is necessary.”

Discussion:

"However, reduction or complete absence of tau did not prevent fibril-induced α-synuclein inclusion formation in primary hippocampal neurons or in vivo, in the cortex, amygdala, hippocampus, or SNc at either early or late time points after fibril injections, consistent with a previous report (Bassil F et al. 2021).”

3) Results show that reductions in tau levels do not influence the formation of phosphorylated alpha-synuclein inclusions in vitro or in vivo - however this is misstated in the abstract, “However, tau heterozygous and tau knockout mice did show a reduction in fibril induced α-synuclein inclusions formation in primary cortical neurons or after intrastriatal injections of fibrils at 1.5 month or 6 months later.” This needs to be corrected.

We have corrected this statement and thank the reviewer for pointing out this mistake.

4) In the introduction: “The effects of tau reduction in synucleinopathy models...” probably should state in something like “neurotoxic or synculeinopatic PD models” as authors in previous sentence mention MPTP and 6OHDA models.

We have made these edits according to the suggestion of the reviewer and thank the reviewer for helping to make the introduction easier to read.

5) In Materials and Methods Animals: the authors sufficiently described transgenic animals (which is not always done) but they should also give reference allowing the interested reader to find details about these animals

The full description of the mice is in preparation by Dr. Erik Roberson’s lab. The constructs flox exon 1 so the resulting deletion is essentially identical to that in the Dawson et al 2001 knockout mice . We have edited the methods accordingly.

6) In Materials and Methods Preparation of Fibrils: this section is very well described. The authors could also clarify how were PFFs kept between sonication and injections (they only state that during injections, they were kept at RT, but do not say if sonication was done each day, or maybe batches were stored frozen).

We are happy to provide any details that will help others replicate our work. We added the following statement to the methods:

"Twenty-two μ;L aliquots were stored at -80{degree sign}C. On the day of injection, the fibril aliquots were thawed and sonicated...”

and

"We have found the fibril fragments generated using this procedure remain stable for at least 3 days at room temperature.”

7) In Materials and Methods Preparation of Fibrils: Dynamic light scattering is very useful in checking between batch variation of sonicated fibrils, but as far as we know absolute quantification of the size of elongated particles is troublesome with this method (it work best for round particles). Are authors sure they can state the 60nm size of fibrils from DLS measurement? If this is approximate, please mention it.

We added the following statement to the methods: “Although this method is optimized for round particles and only approximates the sonicated fibrils, this method ensures sufficient fragmentation of the fibrils (Froula et al. 2019) which is essential for the fibril model to induce sufficient formation of α-synuclein inclusions.”

We also cited our reference, (Froula et al. 2019) in which we collaborated with the biophysicist, Dr. Nunilo Cremades and used techniques such as transmission electron microscopy, atomic force microscopy, fourier transform infrared microscopy, circular dichrosism and analytical ultracentrifugation to characterize the fragments.

8) In Materials and Methods: Authors should provide specific RRIDs of all antibodies.

We have added the RRIDs of all the antibodies.

9) In Materials and Methods Behavior testing: authors mention “tube test,” but it is not later reported.

We removed the mention of the tube test.

10) In Materials and Methods Behavior testing: authors write about main experiments done after 6 months, but should also mention here experiment done 1.5 months after PFFs injections.

We added the 1.5 month time point to both the behavior testing and immunohistochemistry/immunofluorescence sections of the materials and methods.

11) In Materials and Methods Behavior testing: what was the open field container size? What was illumantion (lux)? This is important if open field is used as anxiety measure.

We added the following to the materials and methods section: “Mice were placed in an open field apparatus that was 43cm X 43cm with 30cm high walls. To prevent bias from external factors, the walls were opaque such that mice could not view the external environment and a white noise generator was turned on for the entirety of the testing day. The lighting in the room was the overhead fluorescent lights.” The lights were not dimmed to reduce anxiety.

12) There are two results sections with the same title: “Tau reduction does not prevent inclusion formation or neurodegeneration at 6 months after injection of α-synuclein fibrils.” This needs to be corrected to reflect the 1.5 month time point instead. It is suggested that the results of inclusion formation in vivo at both time points be combined in the results section followed by the behavioral data from both time points, with the earlier time point presented first. In addition, a description of the 1.5 month euthanasia time point does not appear in the methods along with number of mice in this portion of the study.

We have corrected the mistake in our section headings. We agree that the paper may flow better if we lump the 1.5 and 6 month data together. However, the 1.5 month experiments were only performed with WT and HET mice and not KOs. This is because reduced tau and not absence of tau would more likely have translational potential. Also, we had bad luck generating enough knockout mice to have power for statistical significance in a new cohort. We did check the Mendelian inheritance of our WT, HET and KO mice and it follows the predicted patter of 25%, 50% and 25%. In summary, we believe the current way our manuscript is structured allows us to explain that we repeated some experiments in a new cohort of just WT and HET mice.

13) In Fig1D and other. The authors quantify together both somal and neuritic aggregates. For in vivo experiments it’s difficult to distinguish them unequivocally. But for in vitro experiments it’s possible to separately count the number of neurons (neuN positive cells) harboring aggregates in cell soma. This would be interesting considering Tau axonal localization interaction occur in neurites. Could the authors provide such quantification?

The reviewer is correct that one of the advantages of primary neuron experiments is better spatial resolution. We re-analyzed the abundance of aggregates focusing on the neuritic aggregates only. The primary neurons were co-stained with antibodies to p-α-synuclein and NeuN which reveals neuronal nuclei. We used Fiji software to delete all pSyn signal juxtaposed to the nuclei. Fiji software was used to calculate the percentage of area occupied by neuritic p-α-synuclein or neuritic + soma p-α-synuclein (included in Figure 1). As seen in the graphs below, removal of soma p-α-synuclein reduces the % area occupied by p-α-synuclein signal. However, there the differences in the abundance of neuritic aggregates is not significantly different between WT, HET or KO neurons.

14) Figure 1: The descriptor of red to indicate the vGlut in B should be modified as perhaps a better descriptor would be pink as it looks more pink in the version I’m reviewing. Conceptually, it might make more sense to present the data for the

lack of difference in inclusion formation between the three tau genotypes (D) before discussing the Homer vGLUT1 colocalization data. Also, the spacing of the lower fibril panel in B could be optimized for clarity that it is part of B.

We rearranged the figure and results section so that the data for the lack of difference in inclusion formation comes before the analysis of synapses. We also changed the descriptor of vGLUT1 to magenta. We hope that by rearranging the figure, the data is easier to follow.

15) Figure 2: It would be helpful to see primary antibody deleted examples or blocking peptide controls in both the case of non-expanded and expanded tissue to convincingly demonstrate not background immunofluorescence. Fig 2B - insets with bigger magnification would be beneficial.In Figure 2D it looks like for both the cortex and striatum there is a trend toward an increase in a-syn expression with KO of tau, is the experiment powered appropriately to determine whether this is an actual difference?

We have repeated the experiment and included a without primary control, which is included in Figure 2. We also included a panel with a larger magnification to more easily visualize the overlap of tau/α-synuclein/vGLUT1.

We used G*Power to calculate the sample size needed to achieve significance with 80% power and an α of 0.05. To achieve a signification difference in α-synuclein expression among WT, HET and KO mice, would require a sample size of 9 per group. We therefore stated in the results section that although there appears to be a trend toward an increase in α-synuclein expression in the cortex and striatum, our data are not powered to detect a significant difference.

16) Figure 3: The addition of higher magnification inserts would allow for better appreciation of the inclusions. Same suggestion for Figure 6. Also, consider y axis break for graphing the dentate and CA1-CA3 graphs.

We have added higher magnification inserts as suggested by the reviewer to Figure 3 and Figure 6 and hope this helps with better visualization of the inclusions.

Unfortunately, because one heterozygous mouse showed more abundant pathology than the others, we cannot modify the y-axis. The reviewer may have not been able to see the counts from this one mouse because the symbols were in gray, so we change the symbol colors to black for easier visualization. The graphs are represented as whisker plots rather than bars because the hippocampal aggregate data does not fit a normal distribution.

17) Figure 4: Please present actual TH positive counts either in the text or graphed instead of percentages in the figure. Are there any differences in TH neuron number due to the genotype? The image of the tau KO monomer mouse condition looks like the SN may have fewer neurites/neurons - is this image representative? Also, Authors compare monomer to fibril injected animals. However in text they state: “α-Synuclein monomer-injected animals showed no loss of TH-positive neurons in the SNc” how this is know when there was no “sham/untreated” group and injections were bilateral?

Figure 4 was modified to include actual TH positive counts. Because these mice received bilateral injections, counts of both the right and left SNc were included. There were no differences in TH-positive neuron counts within genotype in either α-synuclein monomer injected mice or α-synuclein fibril mice. There was no difference in the amount of TH-positive neuron loss in the SNc of fibril injected mice relative to monomer injected mice comparing wild type, tau heterozygous, and tau knockout mice (Figure 4A,B).

We removed the statement that “"α-Synuclein monomer-injected animals showed no loss of TH-positive neurons in the SNc”.

18) The authors don’t discuss genotype×treatment interaction they mention for figure 5B. “Statistical analysis showed the interaction of genotype and treatment to be significant, as well as genotype alone, but not treatment independently (Figure 5B)

We revised this statement as follows: “Statistical analysis using two-way ANOVA and post-hoc tests revealed as significant difference between monomer injected wild type and monomer injected knockout mice, (Figure 5B); tau knockout monomer-injected mice froze significantly less than wild type monomer-injected mice.”

19) Second section results section, first sentence, missing a word:

"α-Synuclein expression and fibril-induced α-synuclein inclusion formation depends synapse formation (Murphy et al., 2000; Volpicelli-Daley et al., 2011).”

The reviewer has a good eye. We revised this sentence as follows: α-Synuclein expression and fibril-induced α-synuclein inclusion formation depends on synapse formation (Murphy et al., 2000; Volpicelli-Daley et al., 2011).

20) Journal guidelines should be checked for visual abstract, RRIDs and statistical table. There is no statistical methods section presented.

We have added a statistical methods section and all RRIDs.
