# Supplementary figures and images for: Templated α-Synuclein Inclusion Formation Is Independent of Endogenous Tau
Source: eNeuro. 2021 Jun 16;8(3):ENEURO.0458-20.2021. doi: 10.1523/ENEURO.0458-20.2021 (PMC8213444; doi:10.1523/ENEURO.0458-20.2021)

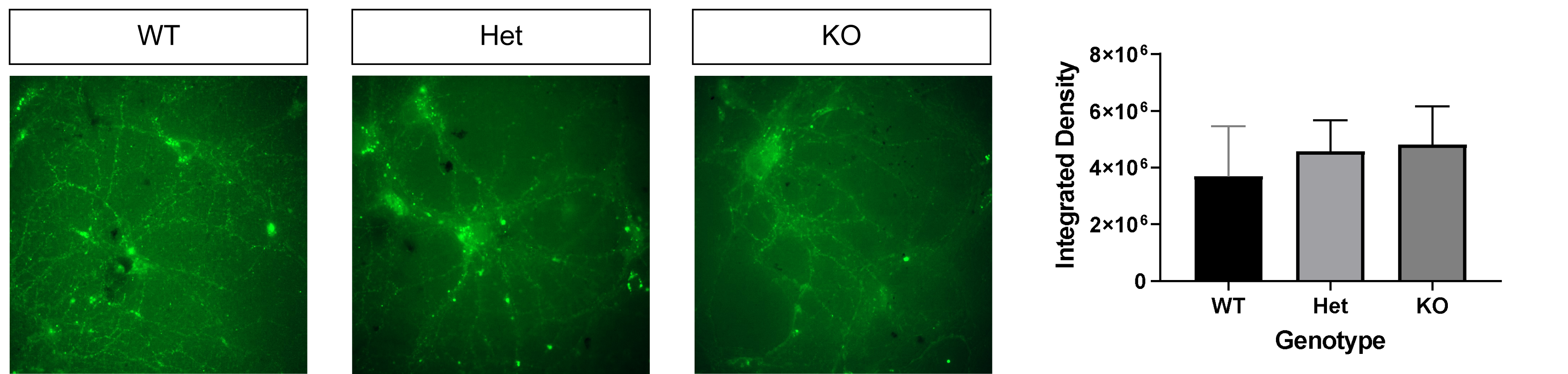

Supplement: Extended Data Figure 1-1 — Internalization assay was completed as described previously (Karpowicz et al., 2017; Froula et al., 2019). Primary cortical neurons were cultured on poly-d-lysine coated MatTek dishes at a density of 5 × 10^5^ cells per plate as described above. At 7 DIV, sonicated fibrils were diluted in cold imaging media (136 mm NaCl, 2.5 mm KCl, 2 mm CaCl2, 1.3 mm MgCl2, 10 mm glucose, and 10 mm 4-(2-hydroxyethyl)-1-piperazineethanesulfonic acid) for a final concentration of 1 μg/ml and added to cultures. Plates were then incubated on ice for 30 min to allow the fibrils to bind to the plasma membrane. The cells were then incubated at 37°C for an additional 30 min to allow internalization. Images were captured using a Zeiss Axio Observer Z1 with Colibri LED illumination. Fluorescence of extracellular tagged fibrils was quenched with addition of 1 mg/ml (1 mm) trypan blue in PBS. Images were captured at an excitation of 470 nm for internalized Alexa Fluor 488-labeled fibrils and trypan blue labeled neurites at 560 nm. The images were analyzed using ImageJ by manually defining a minimum background threshold for the images After the threshold had been set for each group, the integrated density for each image was generated. Results of two independent experiments, WT (N = 2 independent mice), tau heterozygous (N = 2 independent mice), tau knock-out (N = 3 independent mice). Kruskal–Wallis test, H = 0.6935, p = 0.7070. Download Figure 1-1, TIF file. [file enu-eN-NRS-0458-20-s02.tif]

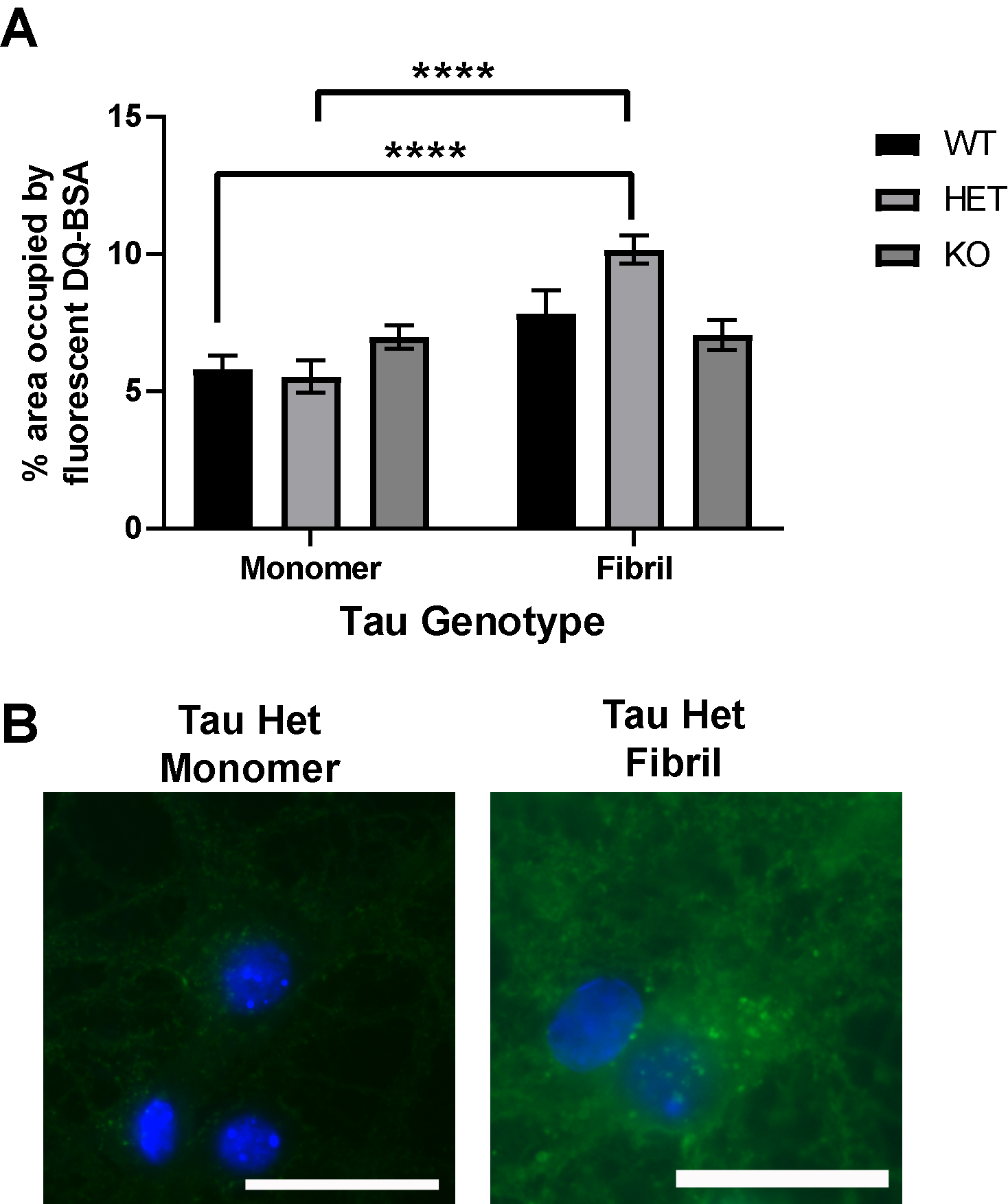

Supplement: Extended Data Figure 1-2 — On DIV7, neurons were treated with α-synuclein fibrils or monomer (1 μg/ml). On DIV14, neurons were treated with DQ-BSA Red (Invitrogen) substrate (images are pseudo-colored green for easier visualization). DQ-BSA Red was solubilized in PBS to a stock concentration of 1 mg/ml and sonicated with a probe tip sonicator for 5 s with a 1-s pulse at 20% amplitude, followed by syringe filtering with a 0.22-μm membrane. Neurons were incubated with 25 μm DQ-BSA Red solution for 2 h at 37°C. Neurons were then fixed with 4% PFA and incubated in Hoechst before mounting the coverslips with Prolong Gold (Invitrogen). For each treatment and genotype, 15–30 random fields were captured using a Zeiss widefield microscope. Following imaging, ImageJ analysis software was used to quantify and analyze DQ-BSA substrate data. After manually drawing the neuronal region of interest containing bright fluorophores, the % area occupied by the puncta was determined using ImageJ. WT (N = 2 independent mice), tau heterozygous (N = 2 independent mice), tau knock-out (N = 3 independent mice). Data were analyzed by two-way ANOVA. Interaction: F(2,268) = 7.2, p = 0.0009; treatment: F(1,268) = 22.1, p < 0.0001; genotype: F(2,268) = 1.678, p = 0.1886; ****p < 0.0001. Scale bar: 50 μm Download Figure 1-2, TIF file. [file enu-eN-NRS-0458-20-s03.tif]

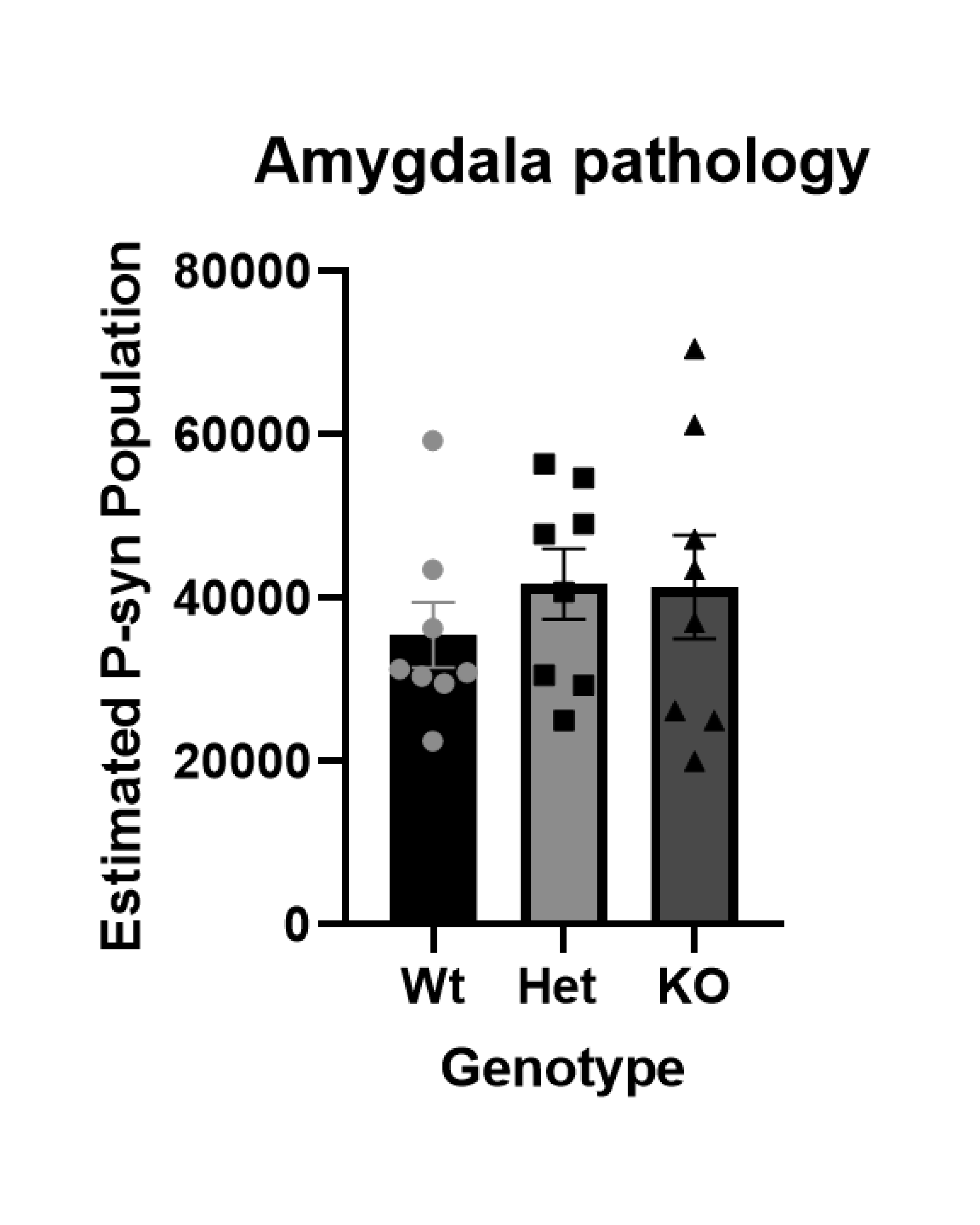

Supplement: Extended Data Figure 3-1 — Unbiased stereology was performed to count p-α-synuclein aggregates in the basolateral amygdala in mice six months after fibril injections. Unbiased stereological analyses were conducted on a fluorescence microscope using an optical fractionator probe (Stereo Investigator software, Stereology Resource Center) on sections stained for phosphorylated synuclein. For stereology of the basolateral amygdala, a 4× objective was used to identify the borders, then a 40 × 0.75 NA objective was used to count p-syn. Sections covered the entire BLA amygdala and were equally spaced 200 μm apart. A total of four to six sections per animal were quantified. Serial sectioning was used to identify sections between bregma coordinates –2.18 to –0.82 mm. The optical dissector height was 22 μm, and the distance between the counting frame was 50 × 50 μm. The grid size was 150 × 150 μm. The counting variability was measured with the Schmitz–Hof CE and was 0.064. One-way ANOVA. Interaction: F(2,21) = 0.4911, p = 0.618; treatment. N = 8/group. Download Figure 3-1, TIF file. [file enu-eN-NRS-0458-20-s04.tif]
